# Modulating the complement system through epitope-specific inhibition by complement C3 inhibitors

**DOI:** 10.1016/j.jbc.2025.108250

**Published:** 2025-01-31

**Authors:** Zhidong Chen, Mingshuang Wang, Wenqian Duan, Yi Xia, Huiqin Liu, Feng Qian

**Affiliations:** 1 School of Pharmaceutical Sciences, Beijing Frontier Research Center for Biological Structure, and Key Laboratory of Bioorganic Phosphorus Chemistry & Chemical Biology (Ministry of Education), Tsinghua University, Beijing, PR China; 2 Quaerite Biopharm Research Co., Ltd., Beijing, PR China

**Keywords:** complement system, complement C3, epitope-specific targeting, process-specific bioassay, structure-activity relationship (SAR)

## Abstract

As an integral part of the innate immune system, the complement system is a tightly regulated proteolytic cascade, playing a critical role in microbial defense, inflammation activation, and dying host cell clearance. Complement proteins are now emerging as subjects of intense research and drug development, since dysregulation of the complement system plays a critical role in several diseases and disorders, such as paroxysmal nocturnal hemoglobinuria (PNH) and geographic atrophy (GA). Within the complement cascade, complement C3 is the central component, situated at the convergence of all complement activation pathways, rendering it an attractive target for complement-related diseases. However, due to the complicated structure-activity relationship (SAR) of C3, elucidating the mechanisms of C3 inhibition on diverse epitopes is the basis for the rational design of C3-targeted therapeutics. Here, we have developed a set of comprehensive biochemical assays that are tailored to the specific steps within the complement cascade, allowing for a thorough understanding of the pharmacological consequences of different C3 inhibitors at each stage. Utilizing three model inhibitors (MIs) with different epitopes, we found that inhibition of MG4/MG5 domains has potent inhibition efficacy across all the complement activation pathways by interrupting C3-C3 convertase interaction, while inhibition of C345C domain displays a bias over the Alternative pathway (AP) inhibition by impairing AP C3 proconvertase formation. This study elucidates the intricate impact of C3 inhibition by targeting different epitopes, offering valuable insights into understanding the mechanism and facilitating the rational design of C3-targeted therapeutics.

## Graphical Abstract

**Figure:**
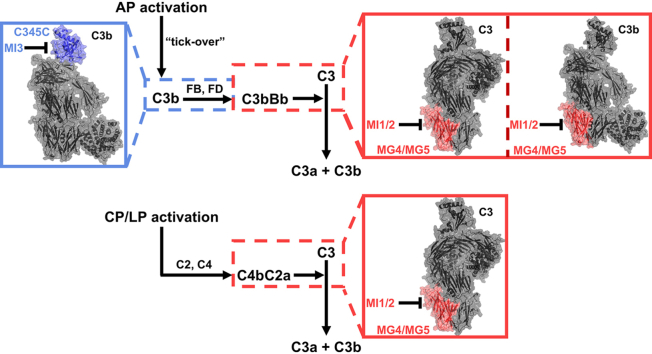

The complement system is a significant part of the innate immune system and primarily serves as a swift host defense system through opsonin-mediated phagocytosis, immunomodulatory activities, and lysis of invading pathogens (1). The complement system is also increasingly acknowledged for its role in maintaining tissue homeostasis and host immunosurveillance, through the clearance of abnormal, damaged, and dying host cells (2). The complement cascade is stringently controlled by a number of negative regulators to prevent inappropriate activation and subsequent damage to healthy host cells (3). Dysregulation and over-activation of complement cascade have been implicated in several diseases, including Paroxysmal Nocturnal Hemoglobinuria (PNH) (4), wet Age-related Macular Degeneration (wAMD) (5), and Geographic Atrophy (GA) (6, 7).

Involving around 50 different proteins, the complement system operates as a meticulously regulated protein network that becomes activated in a cascade-like fashion, with three distinct activation pathways: classical pathway (CP), lectin pathway (LP), and alternative pathway (AP) (8). Three activation pathways converge the formation of C3 convertase (CP/LP: C4bC2a, AP: C3bBb) and the cleavage of complement C3, leading to the release of opsonic fragment C3b and anaphylatoxin C3a. Anaphylatoxin C3a has broad and potent immunomodulatory functions, including inflammatory activation and immune cell recruitment. C3b can be covalently deposited on the surface, forming the new C3 convertase after the action of complement Factor B (FB) and Factor D (FD). The addition of C3b to the existing C3 convertases results in C5 convertase formation (CP/LP: C4bC2a(C3b)_n_, AP: C3bBb(C3b)_n_), which is responsible for cleaving the C5 and participating in the terminal effect of the complement system, including the immune effects of the anaphylatoxin C5a and the lysis as well as the proinflammatory signaling activation effects of Membrane Attack Complex (MAC) (9).

As discussed earlier, C3 and its fragments serve multiple purposes across the entire complement cascade. C3 is the most abundant serum complement protein, acting as a convergence point of activation pathways, fueling the amplification of the complement response (10). C3b, the vital product of C3 conversion, retains the vast majority of the domains of C3 except for C3a but has significant conformational changes compared with intact C3. C3b not only amplifies the complement reaction through the formation of C3 and C5 convertase but also acts as an effector mediating opsonization. It has also been shown that excess C3b deposition can induce conformational change and activation of C5 protein even in the presence of C5 inhibitors (11).

In line with the central role of C3 and its fragments in complement activation, amplification, and immunological effects, the development of drugs targeting C3 for the treatment of complement-related disease is in vogue (12). So far, Empaveli (pegcetacoplan injection) (13) and Syfovre (pegcetacoplan intraocular injection) (6) have been successfully launched for the treatment of PNH and GA, respectively, both of which used an anti-C3 cyclic peptide from the compstatin family as the active motif. Inspired by these clinical findings, there are numerous compounds targeting C3 being evaluated in the clinical-stage or preclinical studies for the treatment of various diseases (14). As such, it is worth considering the biological mechanism and design principles of C3 inhibitors.

Theoretically, C3 inhibition will have comprehensive inhibition effects in the whole complement cascade (15, 16). However, there are notable variations in the effectiveness of inhibition and clinical benefits among different C3 therapeutics. Empaveli (pegcetacoplan injecting) (13) and Syfovre (pegcetacoplan intraocular injection) (6) have met their primary endpoints in clinical trials, while another C3 targeting monoclonal antibody NGM621 failed in the phase 2 study for GA treatment due to lack of efficacy. The varying effectiveness of the C3 therapeutics may be partially attributed to the structural and functional complexity of C3, which complicates the design of C3 therapeutics.

C3 as well as C3b will interact with several complement factors (*e.g.*, proteases, receptors, and regulators) and non-complement proteins (*e.g.*, viral and bacterial proteins) through different epitopes, which are responsible for both complement activation and regulation (10, 17). To inhibit the wide-ranging biological effects of C3-mediated complement activation with high potency for optimal clinical benefit, a potent C3 inhibitor should bind the pivotal epitope with high affinity. In addition, there are epitopes in C3b, like MG7/CUB/C345C domain, that are responsible for the endogenous negative regulation of the complement system (18), and the C3 inhibitors targeting these epitopes will have potential safety issues, such as the accumulation of C3b and related complement activation (19). Therefore, comprehending the biological effects of targeting specific epitopes forms the foundation for designing powerful C3-targeting therapeutics.

In this study, we conducted a thorough investigation into the structure-function analysis of two distinct epitopes by employing three different anti-C3 model inhibitors (MIs) and utilizing various newly developed bioassays tailored to the specific processes in the complement cascade. We here established a clear relationship between the C3/C3b epitope and its inhibition effects, which could facilitate the rational development of potent C3-targeting therapeutics.

## Results

### MG4/MG5 domains and C345C domain are the specific binding epitopes for MI1/MI2 and MI3, respectively

**Figure 1:**
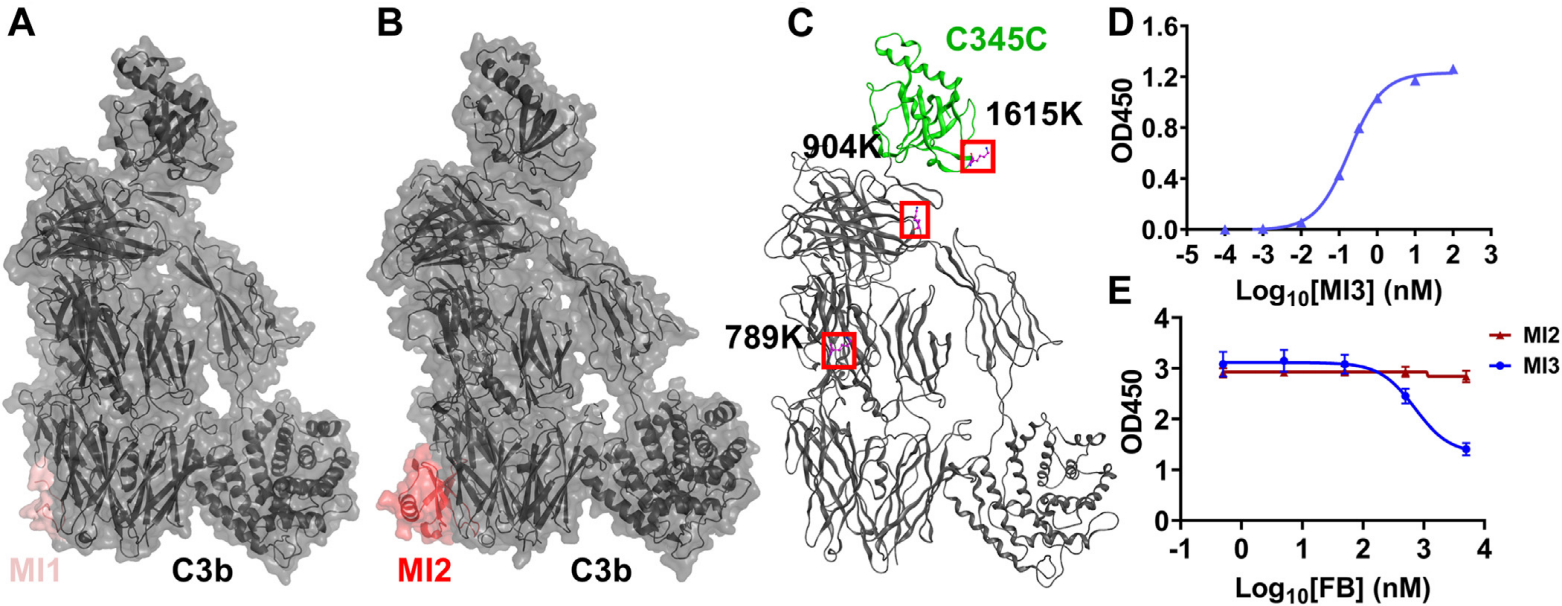
**The binding epitopes of MIs**. *A*, the C3b-MI1 complex structure derived from PDB entry 7BAG. *B*, the C3b-MI2 complex structure derived from PDB entry 7TV9. *C*, the amino acids that formed covalent bonds after cross-linking of the C3b-MI3 complex are marked. *D*, MI3 binds to recombinant C345C domain of C3 and C3b with EC_50_ of 0.19 nM. *E*, FB competitive binding assay. Under the high concentrations of FB, the binding signal of MI3 but not MI2 to C3b is decreased.

To investigate the biological effects of epitope-specific inhibition by C3 therapeutics in the complement cascade, we utilized three anti-C3 MIs. The identities of the three MIs are described in Table S1. Based on the complex structures with C3 and C3b from the previous studies, the MI1 (20, 21) as well as MI2 (22) both target MG4/MG5 domains (Fig. 1, *A* and *B*). The binding affinity of MI1 (21) to C3/C3b was determined by Isothermal Titration Calorimetry (ITC), with K_D_ of 15.6 nM to C3 and 21.3 nM to C3b. The binding affinity of MI2 (22) was determined by surface plasmon resonance (SPR), with K_D_ of 1.59 nM to C3 and 1.11 nM to C3b. The C345C domain within C3 and C3b is a critical functional domain in the complement cascade (23, 24), and therefore, MI3 was included in this study. The binding affinity of MI3 to C3 and C3b was determined by Enzyme-Linked Immunosorbent Assay (ELISA), with EC_50_ of 0.17 nM for C3 and 0.14 nM for C3b (Fig. S2). The epitope of MI3 was confirmed to be located in C345C domain by the Mass Spectrum (MS) and biochemical assay (Figure 1, C-E), along with the *in silico* analysis (Figs. S4 and S5) and the cryogenic Electron Microscopy (cryo-EM) (Fig. S6), as described below.

To determine the epitope of MI3, we first employed the Chemical Cross-Linking MS (CX-MS) to pinpoint the potential amino acids located around the epitope of MI3. In the presence of the chemical cross-linker (bis[sulfosuccinimidyl] suberate, BS3), amino acids 1615K/904K/789K of C3b and 1615K/789K of C3 were identified to form covalent bonds with MI3 (Figs. 1*C* and S3). Given the spatial separation of the crosslinked residues and the small size of MI3, it would be geometrically impossible for all residues to be included within the epitope recognized by MI3, suggesting that some identified crosslinked residues might be the false-positive matches (25).

Biochemical assays were then conducted to further confirm the binding epitope of MI3. The 1615K is located within the C345C domain and 904K is positioned near it, and recombinantly expressed C345C domain was then used to confirm that MI3 binds the C345C domain with EC_50_ of 0.19 nM (Fig. 1*D*). Additionally, FB, which binds C3b at the C345C domain, could inhibit the binding signal of MI3 to C3b in the ELISA-based competitive binding assay (Fig. 1*E*), indicating spatial overlap between the MI3 binding site and the FB binding site in C3b. Moreover, a structure of the C3b–MI3 complex was solved by cryo-EM, providing supporting evidence of the interaction between MI3 and C345C domain as indicated in Fig. S6. Furthermore, molecular docking restricted by all spatially possible combinations of crosslinked residues from CX-MS was performed (Fig. S4). Most docking results were excluded by the properties of interacting with the C345C domain and competing with FB to bind C3b. The docking models consistent with the CX-MS and biochemical results were selected for molecular dynamics (MD) simulation, demonstrating that the interaction between the C345C domain in C3/C3b and MI3 is stable *in silico* (Fig. S5).

Taken together, the CX-MS and biochemical assays, combined with the cryo-ME, molecular docking, and MD analysis, indicated that the binding epitope of MI3 is C345C domain. Consequently, MI3 was utilized to understand the biological effects of this specific epitope in this study.

### Targeting MG4/MG5 domains and C345C domain results in differential inhibition efficacy in CP-mediated hemolysis

In the complement cascade, the LP differs from the CP only in the recognition and initiation units, and they share the same components to cleave C3 and activate related reactions. The C3 therapeutics with the same epitope will have a consistent mechanism of action and potency in both the CP and LP (23). Therefore, we mainly focused on the AP and CP in this study to understand the biological effects of targeting specific epitopes.

**Figure 2:**
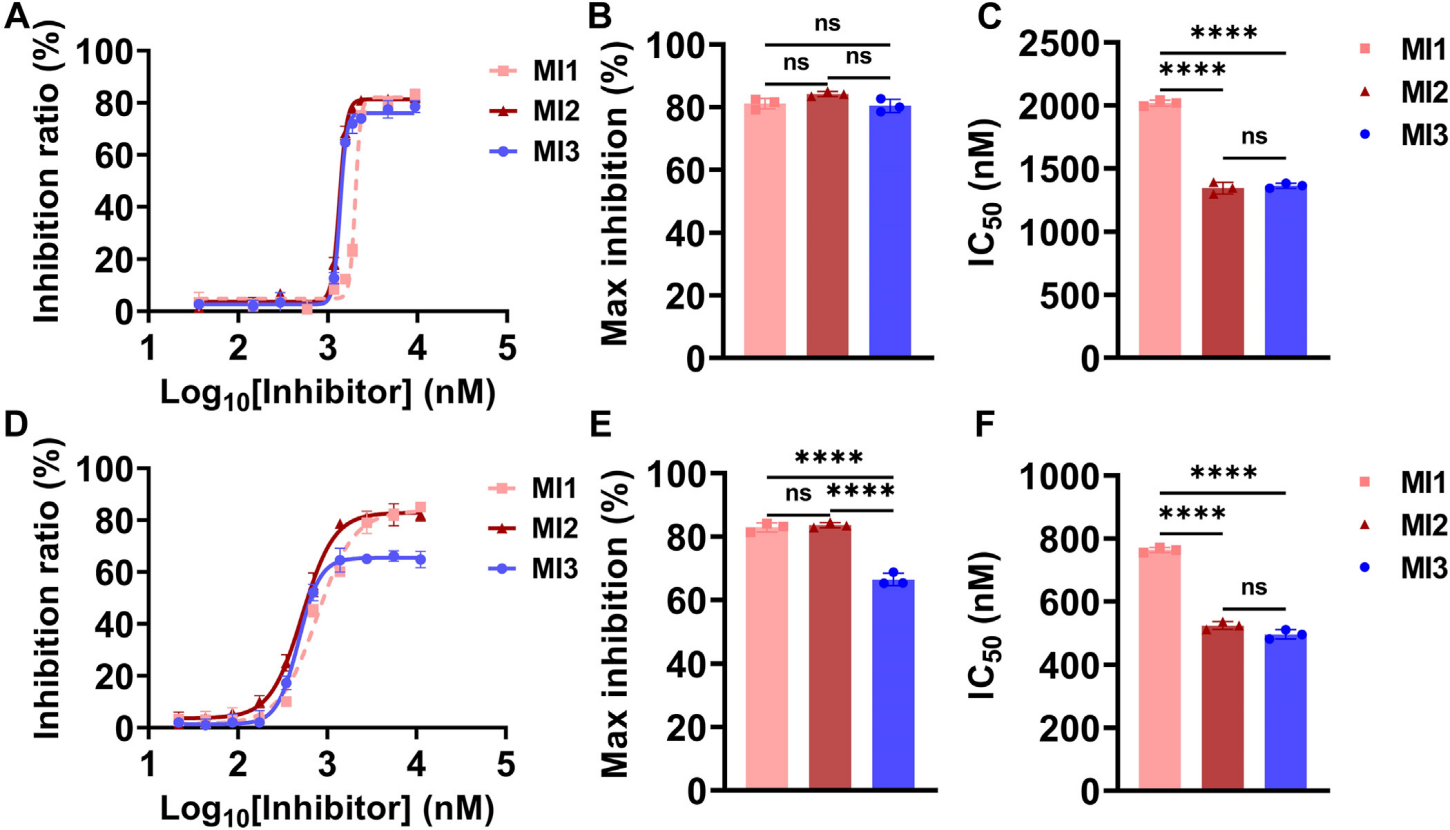
**Targeting MG4/MG5 domains and C345C domain inhibit AP and CP-mediated hemolysis with different inhibition efficacy.** The AP-mediated hemolysis (*A*–*C*) is activated on rabbit red blood cells (rRBCs) and the CP-mediated hemolysis (*D*–*F*) is activated on sheep red blood cells (shRBCs) sensitized by anti-sheep erythrocyte antibodies hemolysin. Data are representative of three independent experiments and are shown as means ± SDs of triplicates. Statistical analyses were performed using GraphPad Prism 9 (https://www.graphpad.com) by one-way ANOVA with Tukey's multiple comparison test. ∗∗∗∗ = *p* < 0.0001, ∗∗∗ = *p* < 0.001, ∗∗ = *p* < 0.01, ∗ = *p* < 0.05, ns = non-significant.

To evaluate the overall complement cascade inhibition activity of the two epitopes, we utilized the hemolysis assay, which is a classical method to reflect the net effect of the complement activation (26). AP and CP-mediated hemolysis were activated on rabbit red blood cells (rRBCs) and antibody-sensitized sheep red blood cells (shRBCs), respectively, with normal human serum (NHS) as the sources of complement activation. All MIs prevent AP and CP-mediated lysis of RBCs in a dose-dependent manner with different inhibition potency, mainly the IC_50_ and maximum inhibition ratio. In AP hemolysis, the three MIs have similar activity, with a maximum inhibition rate of ∼80% (Fig. 2, *A*–*C*). In CP hemolysis, MI3 has a significantly lower maximum inhibition ratio (65%) compared with MI1 (83%) and MI2 (81%) (Fig. 2, *D*–*F*), suggesting that targeting the C345C domain has a bias of the AP while targeting MG4/MG5 domains is a strategy for comprehensive complement inhibition in both the AP and CP. MI2 was more effective than MI1 in two pathways with higher IC_50_, probably stemming from the higher affinity of MI2 to C3 and C3b.

### Targeting MG4/MG5 domains and the C345C domain results in differential inhibition efficacy in CP-mediated C5 cleavage

Since the inhibition of C3 could transmit to its downstream steps in the complement cascade and the hemolysis assay only reflects the net effect, we developed biochemical assays tailored to the different processes in the complement cascade, which is useful for investigating the impact of complement inhibition to gain insight into the mechanism and processes of inhibition by epitope-specific C3 inhibitors.

C3b is a component of C5 convertase (8), and C3 targeting might inhibit C5 convertase formation and C5 cleavage. We speculated that the difference in the max inhibition ratio of CP hemolysis in Figure 2*E* results from the different cleavage degrees of C5. The main component of the MAC is C5b-9, which is the cleavage product of C5 and responsible for the hemolysis of RBC. Therefore, we then investigated the complement inhibition effect of MIs in C5 cleavage by detecting the content of C5b-9 after complement activation.

**Figure 3:**
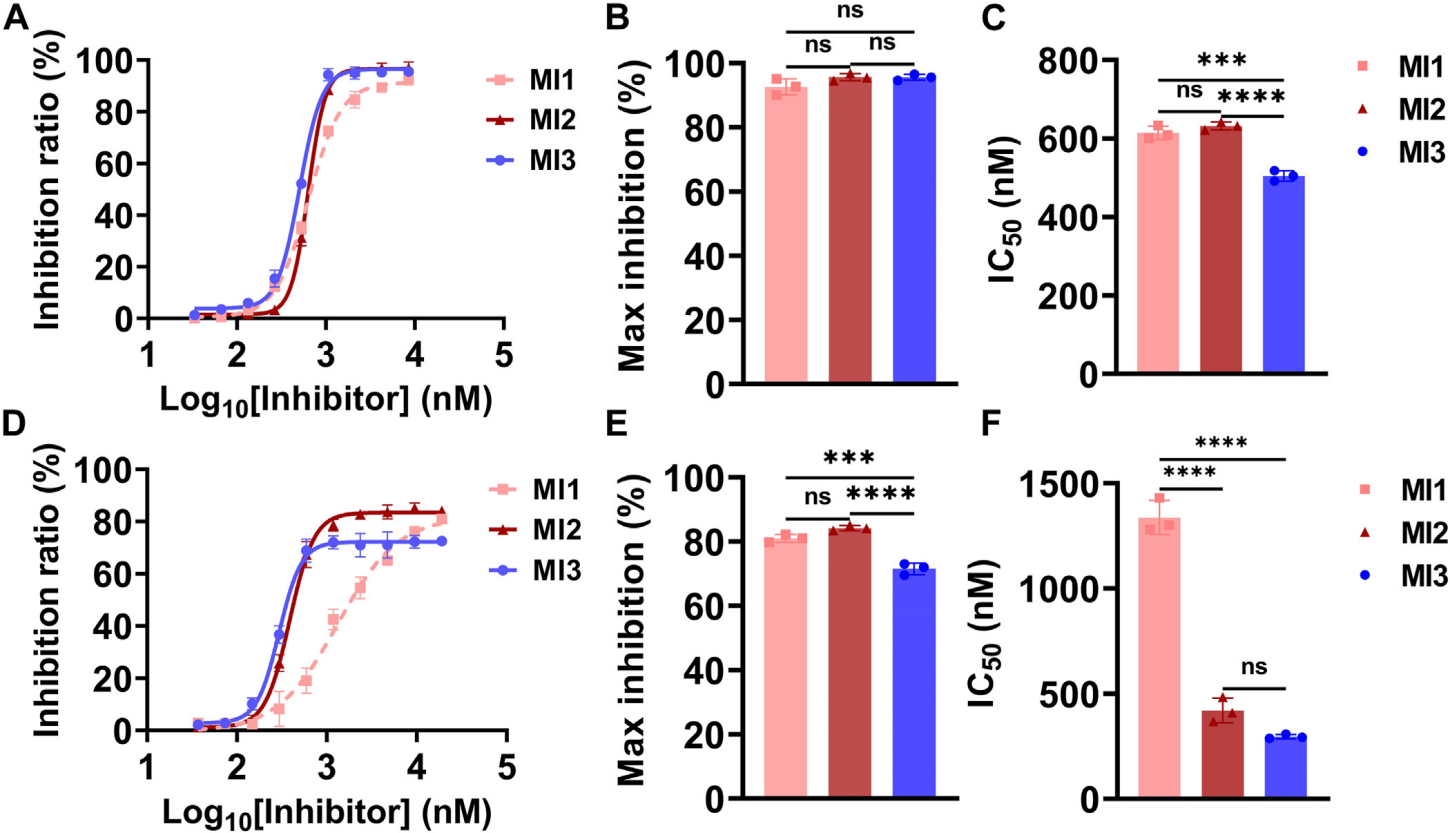
**Targeting MG4/MG5 domains and C345C domain inhibits the AP and CP-mediated C5 cleavage.** C5b-9 deposition was detected on the ELISA plate after complement activation by NHS through AP (*A*–*C*) and CP (*D*–*F*) pathways. Data are representative of three independent experiments and are shown as means ± SDs of triplicates. Statistical analyses were performed using GraphPad Prism 9 (https://www.graphpad.com) by one-way ANOVA with Tukey's multiple comparison test. ∗∗∗∗ = *p* < 0.0001, ∗∗∗ = *p* < 0.001, ∗∗ = *p* < 0.01, ∗ = *p* < 0.05, ns = non-significant.

We initiated the AP or CP of complement cascade on the ELISA plate with NHS activated by zymosan or heat-aggregated IgG respectively, and detected the C5b-9 deposition at increasing concentrations of MIs (Fig. 3). All MIs inhibit C5b-9 deposition in a dose-dependent manner. In the AP, targeting these two epitopes has similar inhibition potency, with a maximum inhibition rate of ∼95% (Fig. 3, *A*–*C*). In the CP, targeting the C345C domain has a lower maximum inhibition ratio (72%) than targeting MG4/MG5 domains (82%) (Fig. 3, *D*–*F*), which is similar to the CP-mediated hemolysis (Fig. 2*E*). We consistently observed a more potent inhibition effect by targeting MG4/MG5 domains than the C345C domain in the CP, suggesting their different inhibition mechanism and potency. In addition, the difference in IC_50_ between MI1 and MI2 in the CP C5 cleavage is larger than that in the CP hemolysis, which might be bioassay-dependent. In the C5 cleavage assay, the complement cascade mainly reacted on the plate, and in the hemolysis assay, the complement cascade mainly reacted in the free RBC. The active motif in MI1 is a PEGylation cyclic peptide, and the PEGylation has been suggested that it might influence the diffusion and target accessibility effects on the plate, which might result in the different activity in these two assays (21).

### Targeting MG4/MG5 domains inhibits the AP and CP-mediated C3 cleavage while targeting the C345C domain selectively inhibits AP-mediated C3 cleavage

The main inhibition effect of C3 therapeutics will result from the complement reaction at the C3 level, therefore we then investigated the physiological inhibition activity and pathway bias of targeting the two specific C3 epitopes in the C3 reaction. The C3 convertases in the AP and CP are assembled *in vitro via* complexation of complement C3b/FB/FD and complement C4b/C2/C1s respectively, and the degree of C3 cleavage is evaluated by quantifying the content of C3a, the C3 degradation product (8). The concentrations of each complement component are set by reference to the corresponding plasma concentration.

**Figure 4:**
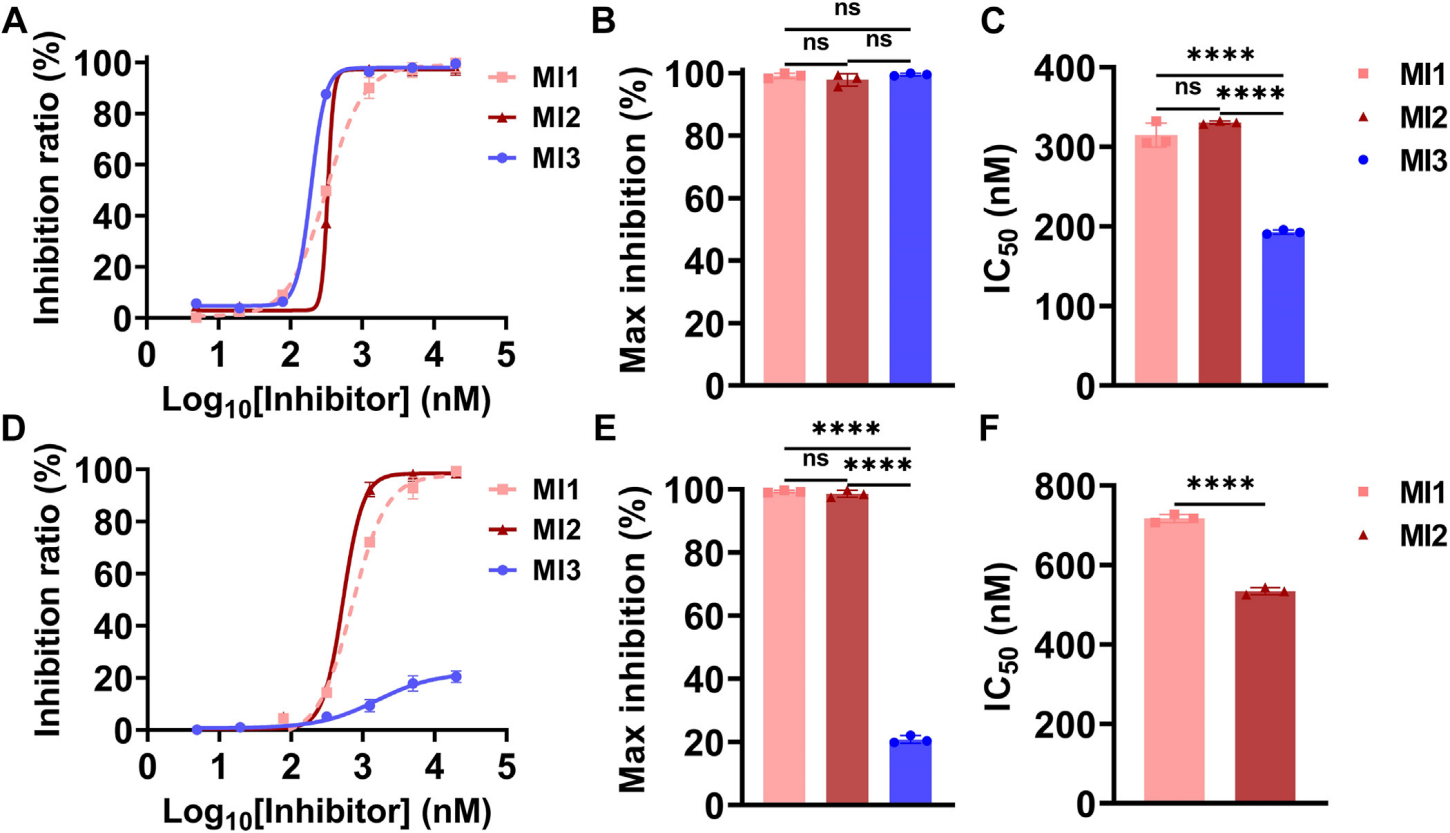
**Targeting MG4/MG5 domains inhibits the AP and CP-mediated C3 cleavage while targeting C345C domain selectively inhibits the AP-mediated C3 cleavage.** Physiological complement inhibition activity and pathway bias of MIs at the C3 level were evaluated by assembling C3 convertases in AP (*A*–*C*) and CP (*D*–*F*) pathways *in vitro*. MI1 and MI2 both inhibit C3 cleavage in the AP and CP, while MI3 displays a significant bias over the AP. Data are representative of three independent experiments and are shown as means ± SDs of triplicates. In (*B*, *C*, and *F*), statistical analyses were performed using GraphPad Prism 9 (https://www.graphpad.com) by one-way ANOVA with Tukey's multiple comparison test. In (*F*) statistical analysis was performed by unpaired *t* test. ∗∗∗∗ = *p* < 0.0001, ∗∗∗ = *p* < 0.001, ∗∗ = *p* < 0.01, ∗ = *p* < 0.05, ns = non-significant.

In the AP C3 convertase (C3bBb) system (Fig. 4, *A*–*C*), all MIs demonstrate complete inhibition, with MI3 (IC_50_: 195 nM) being more potent than MI1 (IC_50_: 314 nM) and MI2 (IC_50_: 330 nM). In the CP C3 convertase (C4bC2a) system (Fig. 4, *D*–*F*), MI1 (IC_50_: 717 nM) and MI2 (IC_50_: 529 nM) exhibit complete inhibition (100% inhibition), but the inhibition effect of MI3 is poor (20% inhibition).

Targeting MG4/MG5 domains completely inhibits the cleavage of C3 by both AP and CP C3 convertases, resulting in the high efficacy of MI1/MI2 in the hemolysis assay of two pathways. In addition, MI1/MI2 exhibit a slight bias over the AP at the C3 level, with better IC_50_ in AP than in the CP under the plasma concentration.

Compared with the hemolysis assay, targeting the C345C domain shows a more pronounced bias over the AP at the C3 level. Targeting C345C domain with MI3 barely inhibited CP-mediated C3 cleavage (Fig. 4, *D*–*F*), and consequently, the maximum inhibition ratio in CP hemolysis is lower than that of targeting MG4/MG5 domains using MI1/MI2 (Fig. 2). In addition, in the CP, there is a significant disparity between the maximum inhibition ratio of targeting C345C domain in the hemolysis (Fig. 2, *D*–*F*) and C3 cleavage assays (Fig. 4, *D*–*F*), suggesting that targeting C345C domain might have inhibited the CP mostly through other processes in the complement cascade. Based on the C5 cleavage assay (Fig. 3, *D*–*F*), targeting the C345C domain has no effect at the C3 level and only inhibits the CP-mediated hemolysis by the function of C3b in the CP C5 convertase.

**Figure 5:**
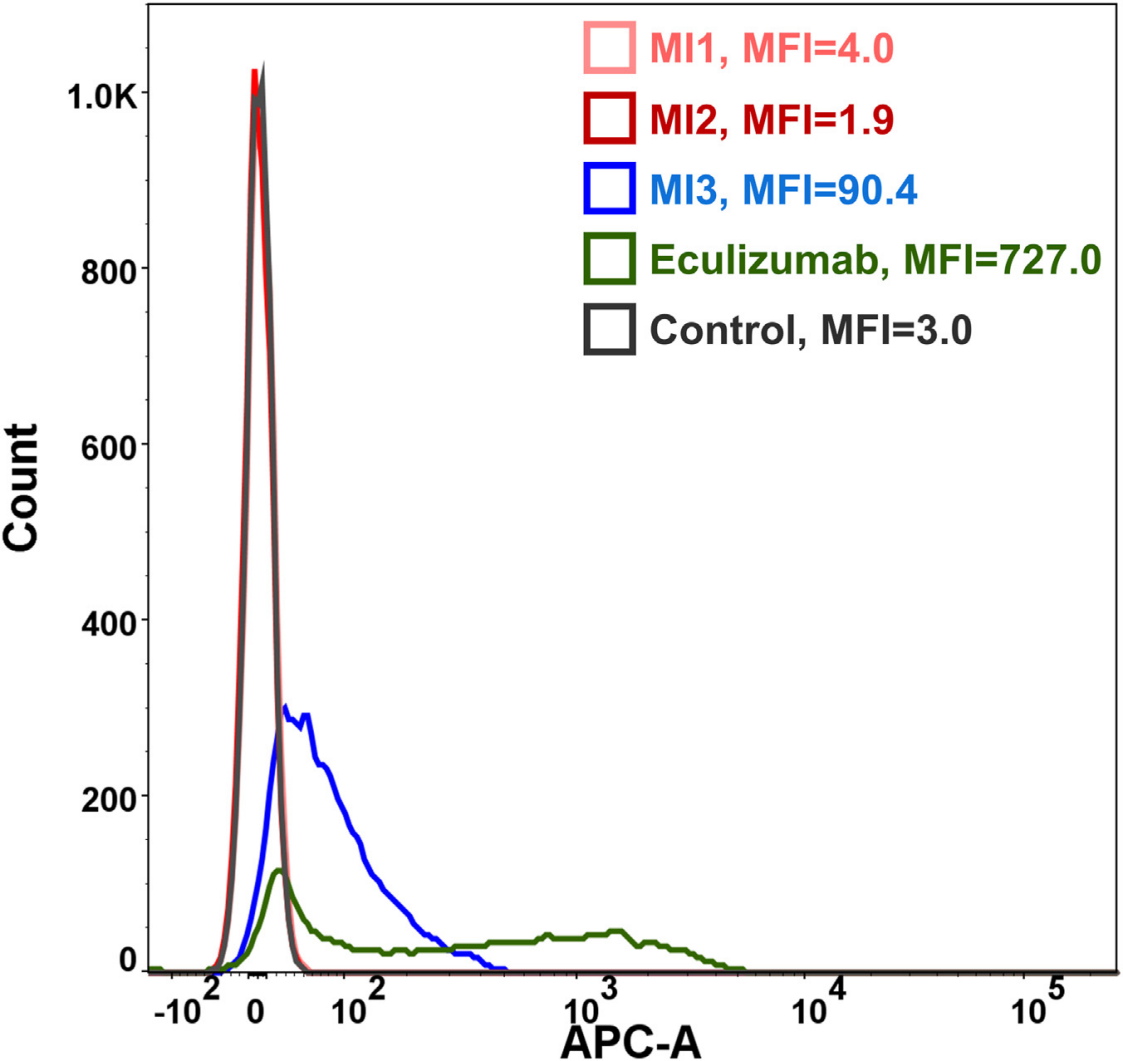
**Targeting MG4/MG5 domains and C345C domain inhibits the C3b deposition on the rRBCs**. The signal of APC-A comes from the C3b deposition on the rRBCs surface. “Control” is the system without NHS and MIs. The APC-A measures the level of C3b deposition. MFI, Mean Fluorescence Intensity.

C3b is another degradation product of C3, having significant physiological functions, and is also a causative component of complement-related diseases such as PNH (16, 27) and GA (28). After activation of the complement cascade, nascent C3b will be deposited on the cell surface, triggering phagocytosis (17). To assess the impact of epitope-specific inhibition on the C3b deposition, we conducted an analysis of C3b deposition levels on the surface of rRBCs following complement activation in the presence of MIs or eculizumab (Fig. 5). Eculizumab, the FDA-approved anti-C5 IgG, could block the terminal complement pathway to prevent lysis of rRBCs without inhibition of the C3 cleavage. As shown in Figure 5, upon incubation with NHS and eculizumab, the residual rRBCs have a much higher level of C3b deposition with an MFI value of 727.0 in the absence of C3 inhibitors. MI1 and MI2 can fully inhibit the C3b deposition, showing a background C3b staining similar to the control group with rRBCs only. Unlike MI1 and MI2, MI3 is unable to completely inhibit C3b deposition, which indicates that distinct inhibition mechanisms at the C3 level are at play when targeting the C345C domain. These results indicated that to achieve complete inhibition of the C3b deposition, it is more effective to target the MG4/MG5 domains rather than the C345 domain.

### Targeting the C345C domain demonstrates AP bias over CP by impairing AP proconvertase C3bB formation

**Figure 6:**
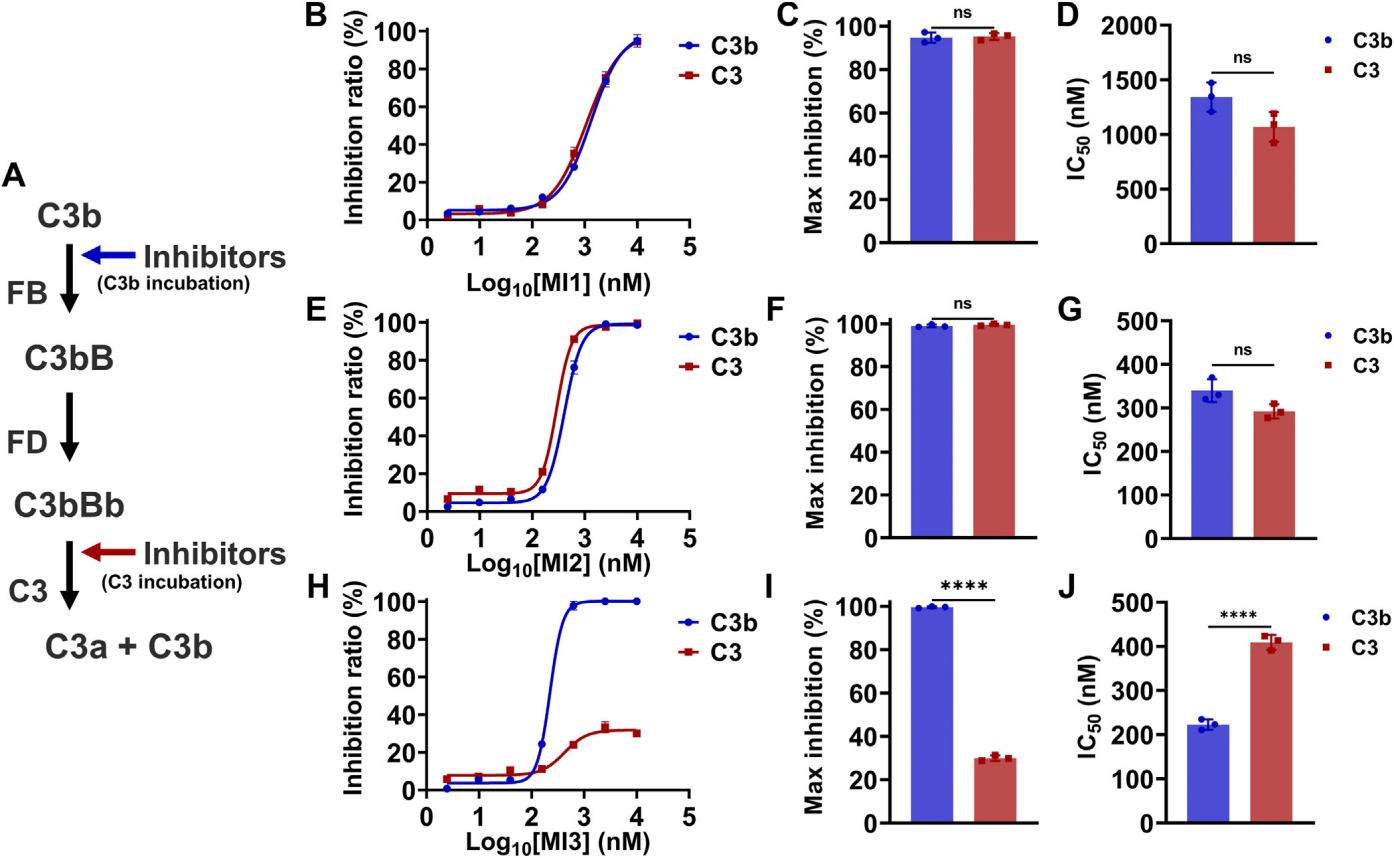
**Targeting MG4/MG5 domains inhibits C3 substrate cleavage and targeting C345C domain inhibits AP proconvertase C3bB formation.***A*, the workflow of C3 convertase formation and C3 cleavage. The inhibition activity of MI1 (*B*–*D*), MI2 (*E*–*G*), and MI3 (*H*–*J*) in the “C3 incubation” and “C3b incubation” methods. MI1 and MI2 incubated with C3 inhibit the C3 from cleavage, while MI3 is unable to prevent C3 from cleavage by the pre-existing C3 convertase (C3bBb). Data are representative of three independent experiments and are shown as means ± SDs of triplicates. Statistical analyses were performed using GraphPad Prism 9 (https://www.graphpad.com) by unpaired *t* test. ∗∗∗∗ = *p* < 0.0001, ∗∗∗ = *p* < 0.001, ∗∗ = *p* < 0.01, ∗ = *p* < 0.05, ns = non-significant.

The essential complement processes at the C3 level are the formation of C3 convertase and the cleavage of C3. To analyze the epitope-specific inhibition mechanism, complement C3b, FB, and FD are assembled *in vitro* to form C3 convertases, and MIs are added to the reaction system at different reaction steps (Fig. 6*A*). In “C3b incubation”, MIs are first incubated with C3b before C3 convertase formation, and complement FB/FD/C3 was added subsequently to the reaction system. In “C3 incubation”, MIs are added to the reaction system after the formation of C3 convertase with complement C3b/FB/FD. The degree of C3 cleavage was also detected by quantifying the content of C3a. The results show that incubating MI3 with C3b could completely and efficiently inhibit the C3 cleavage, but was unable to prevent C3 cleavage by the pre-existing C3b/FB/FD complex (Fig. 6, *H*–*J*). This suggests that targeting the C345C domain by MI3 inhibits complement cascade at the C3 level by inhibiting the formation of AP proconvertase C3bB rather than the interaction between C3-C3 convertase. However, targeting MG4/MG5 domains by MI1 and MI2 demonstrated comparable efficacy when incubated with C3 or C3b (Fig. 6, *B*–*D* and *E*–*G*), suggesting that they might inhibit the C3 cleavage by disrupting the interaction between C3 and C3 convertase.

**Figure 7:**
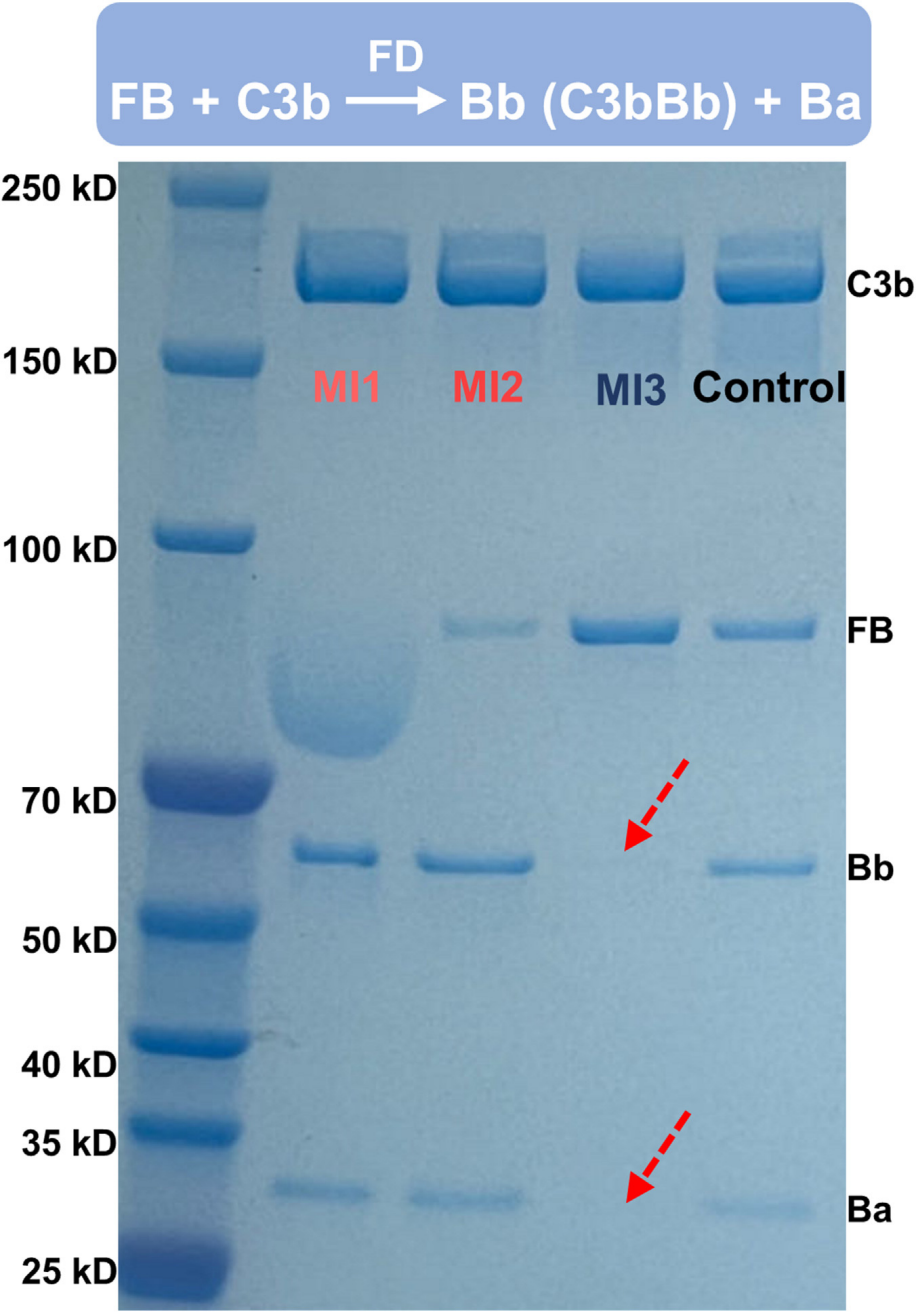
**Targeting the C345C domain inhibits the binding of FB to C3b and AP proconvertase C3bB formation**. MI3 inhibits the cleavage of FB by C3b and FD. The bands of Bb and Ba are the degradation product of FB by C3b and FD.

To further explore the specific mechanism by which MI3 inhibits the C3 cleavage before the formation of the AP C3 convertase and MI1/MI2 show comparable activity in different incubation methods, we analyzed the impact of MIs on C3b-FB interaction by FB cleavage assay (Fig. 7) and FB-C3b competitive binding assay (Fig. 1*E*). In the FB cleavage assay, the cleavage products of FB under the action of C3b and FD, Bb and Ba, are absent when MI3 is present (Fig. 7), implying that targeting the C345C domain by MI3 inhibits the FB-C3b interaction and subsequent cleavage of FB by FD. In contrast, targeting MG4/MG5 domains does not affect the FB cleavage. In the ELISA-based competitive binding as discussed above, the signal value of MI3 decreases at high concentrations of FB (Fig. 1*E*), while the signal value of MI2 remains unchanged, suggesting that FB inhibits the interaction of C3b with MI3 but not MI2. Based on these results, targeting C345C domain using MI3 inhibits the complement cascade by binding to C3b and inhibiting the formation of the AP proconvertase C3Bb, with selective inhibition of AP-mediated C3 cleavage (Fig. 4). Furthermore, the findings suggest that the presence of C3b is essential for the inhibitory effect of MI3, elucidating its comparatively lower efficacy in the C3b deposition assay (Fig. 5). Targeting MG4/MG5 domains effectively inhibits the interaction between C3 and C3 convertases, leading to potent inhibition of C3 cleavage in both AP and CP, without interfering with the formation of C3 convertases. It is worth noting that the binding sites of FB have been determined in the previous study (24), which partially overlaps with the MI3 epitope, forming the structural basis of the inhibition mechanism of MI3 (Fig. S7*B*).

## Discussion

### Structural basis of C3-mediated complement inhibition by targeting specific epitopes

**Figure 8:**
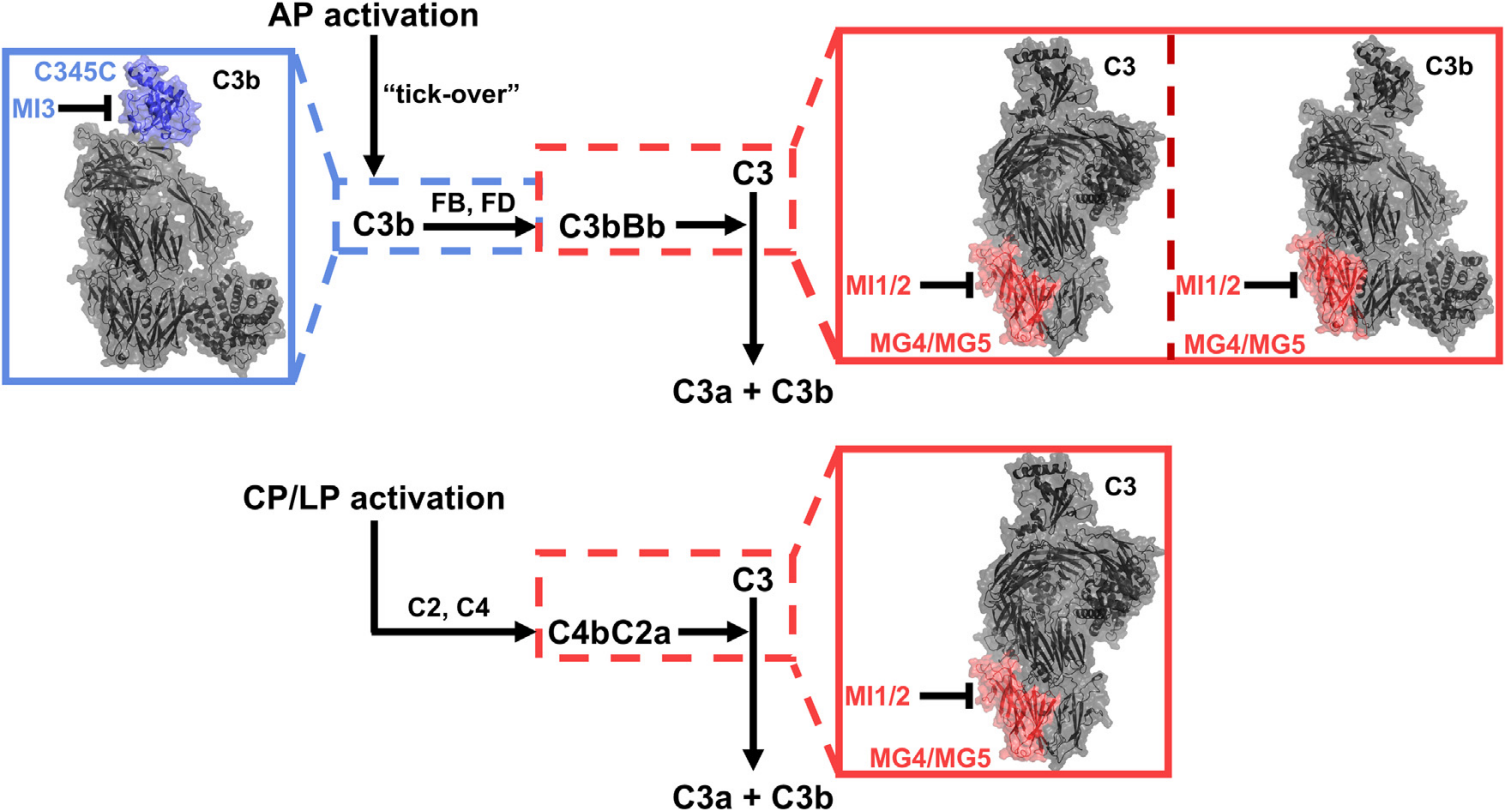
**The processes of inhibition of targeting the C345C domain and MG4/MG5 domains**. In the *blue rectangle* (*left side*), the structure of C3b is shown, highlighted with the binding epitope of MI3, *i.e.*, C345C domain (*blue*). In the *red rectangle* (*right side*), the structure of C3 and C3b are shown, highlighted with the binding epitope of MI1 and MI2, *i.e.*, MG4/MG5 domains (*red*). In AP activation *via* “tick-over” (12) (*top panel*), targeting C345C domain of C3b by MI3 disrupts the formation of AP C3 convertase C3bBb, and targeting MG4/MG5 domains of C3 and C3b by MI1/MI2 inhibits the interaction of C3 and C3bBb. In CP/LP activation (*bottom panel*), targeting MG4/MG5 domains of C3 by MI1/MI2 inhibits the interaction of C3 and CP C3 convertase C4bC2a.

We here performed a comprehensive analysis of biological effects and functional properties after targeting the two specific epitopes by three anti-C3 MIs in the complement cascade. The processes of inhibition of these two epitopes are summarized in Figure 8.

MG4/MG5 domains are located at the interface of the interaction between two C3 convertases and substrate C3 (10, 11, 21), and targeting this epitope can inhibit the cleavage of C3 (Fig. S7*A*), showing high activity across all pathways. In addition, targeting MG4/MG5 domains shows a slight bias towards AP over CP in the C3 cleavage assay, with better inhibition activity in AP. The C3 convertases in AP and CP are C3bBb and C4bC2a, and in these two C3 convertases, C3b and C4b are responsible for C3 binding. MI1/MI2 are specific C3/C3b inhibitors without C4b inhibition, which is the structural basis of the lower inhibition of C3-C4bC2a interaction compared to C3-C3bBb.

C345C domain is located at the epitope responsible for the formation of AP proconvertase C3bB and C5 binding (11, 24), and targeting this epitope can potently inhibit the AP by disrupting the formation of the C3bB and inhibiting the C5 cleavage (Fig. S7*B*). Unlike MG4/MG5 domains, targeting the C345C domain has relatively poor inhibition efficacy in CP-mediated complement activation. Targeting the C345C domain inhibits the CP activation only by inhibiting the binding of C5 to CP C5 convertase (C4bC2a(C3b)_n_) because targeting this epitope does not affect CP-mediated C3 cleavage and only inhibits the binding and cleavage of C5 (11). The inhibitory activity of C5 cleavage (C5 convertase inhibition) by C3 inhibitors has been previously reported (23), which is consistent with our results.

In the complement cascade, complement C3 and C3b share highly similar structures, and many C3 inhibitors are actually dual inhibitors that bind both the C3 and C3b (21, 22, 29), including the compstatin family, NGM621 (data not shown) and the MIs in this study. However, from the perspective of the biological effect in the complement cascade, MI3 is a specific C3b inhibitor, because it can only inhibit the complement cascade through the biological function of C3b rather than C3, including the formation of AP proconvertase C3bB and the activity of C5 convertase. The AP bias of MI3 stems from its functional specificity to C3b with the structural basis of targeting the C345C domain. This specificity is crucial since C3b is solely responsible for activating the AP. In contrast, MI1/2 are C3/C3b dual inhibitors with the inhibition effect on the biological function of both the C3 and C3b, *i.e.*, the interaction of C3 and C3 convertase as well as C3 cleavage.

### Rational design of optimized C3-targeting therapeutics

Inhibition of C3 and its associated complement activation as well as biological functions represents a promising strategy for complement-related diseases. Currently, there are several C3 therapeutics in the preclinical and clinical stages of development (14), but only one anti-C3 motif has been approved for the treatment of PNH (Empaveli, pegcetacoplan injection) and GA (Syfovre, pegcetacoplan intraocular injection). The structural complexity and diverse biological effects of C3, as well as the high systemic concentration (in plasma (14)) and localized concentration (*e.g.*, in ocular (30)) of C3, are the main challenges in the design of potent C3 therapeutics (31). An ideal C3 therapeutics with high clinical benefits should specifically inhibit the critical processes of the complement cascade with high efficacy while preserving the regulation of the complement system and beneficial complement activities. Hence, targeting the most effective epitopes with high affinity is a critical requirement for C3 therapeutics. Therefore, it is crucial to uncover the mechanisms of inhibition and functional characteristics of various epitopes, for the determination of the optimum epitope and the rational design of optimized C3 therapeutics.

Different domains and epitopes of C3/C3b contribute differently to complement activation in the dynamic complement cascade. This complicated structure-activity relationship makes it possible to design differentiated C3 therapeutics by targeting specific epitopes to meet different clinical needs. In this study, we found that even with lower affinity, MI1 and MI2 have more potent biological efficacy than MI3 in the CP by targeting MG4/MG5 domains. In conditions where complete C3 inhibition is preferred, such as GA (32) and PNH (16), the epitopes situated at MG4/MG5 domains are promising for the rational design of optimized C3 therapeutics for two reasons (1): Targeting MG4/MG5 domains can completely inhibit C3 cleavage and potently inhibit C5 convertase activity in both CP and AP (2); Targeting MG4/MG5 domains does not affect the degradation of C3b by Factor H (FH) and Factor I (FI), a critical complement inhibition process, and will not lead to a buildup of C3b (Fig. S8).

Targeting a specific epitope in the C345C domain could partially inhibit the complement cascade without completely shutting it down. Evidence suggests that AP inhibition is sufficient to prevent or mitigate specific disease pathologies, and preserving host defense mechanisms provided by the CP and LP is beneficial (33). This suggests that a partial reduction of complement activity is required to restore the activation-regulation balance, rather than a complete inhibition of the complement system (21, 34). Targeting C345C domain allows MI3 to potently inhibit AP while preserving partial CP activity. This mechanism of action holds potential therapeutic benefits for certain diseases, warranting further investigation (33). However, since the C3b degradation through FH and FI is also inhibited by targeting C345C domain (Figs. S7*C* and S8), the possible C3b buildup could lead to excessive complement activation and increased phagocytosis by immune cells such as monocytes, macrophages, and dendritic cells (19), and consequently, the balance of clinical benefit and safety risk of this epitope should be carefully evaluated.

### Process-specific bioassays are tailored tools to comprehend the action mechanisms of the complement inhibitors

In complement drug discovery, there are currently two typical complement assays, the hemolysis assay and the complement deposition assay (35). The hemolysis assay is a classical method to evaluate the net effect of the complement activation, as RBC lysis occurs only if the MAC assembles on their surface, which is the final step in the complement cascade. By different detection reagents, the complement deposition assay can reflect the inhibition effects of complement inhibitors on the cleavage degree of C3, C4, and C5. However, both the hemolysis assay and complement deposition assay mainly reflect the holistic inhibition potency of complement inhibitors, which are unable to provide accurate process-specific mechanisms.

C3 and C3b have complicated structures with different epitopes that are responsible for distinct processes in the complement cascade, and it is critical to develop bioassays that can reveal the process of inhibition in complement drug discovery for various clinical needs. Therefore, the process-specific bioassays serve as valuable tools for gaining insights into the mechanisms of action of different complement inhibitors and are fundamental to the design of complement-targeted drugs. In this study, we developed process-specific bioassays that can clearly demonstrate the process of inhibition and potency of C3 therapeutics, including the C3 convertase formation assays (Fig. 6) and FB cleavage assay as well as FB competitive binding assay (Figs. 1*E* and 7). These process-specific bioassays make it possible to uncover the molecular mechanistic differences between these C3 inhibitors, that neither binding assays (ELISA, ITC, SPR, *etc*) nor hemolysis assay could reveal. In this study, utilizing these bioassays, we were able to find out that targeting the C345C domain demonstrated strong AP bias over CP by impairing AP proconvertase C3bB formation. Additionally, our findings indicate that MI3 is actually a specific C3b inhibitor instead of a C3/C3b dual inhibitor from the perspective of its biological function. In addition, these bioassays also have implications for the study of other complement targets downstream of C3, such as C5.

During the design of drugs targeting complement proteins for specific diseases, it is crucial to precisely define the desired properties of drug candidates and to incorporate corresponding bioassays in the early stages of drug discovery to streamline the selection process. For example, upon the activation and cleavage of C3, its fragments, including C3b, iC3b, and C3d, will opsonize the red blood cells in PNH patients, which will be the targets for macrophages, leading to the consequent extravascular hemolysis (16). Because both the C3b deposition (extravascular hemolysis) and the MAC formation (intravascular hemolysis) are key disease characteristics in PNH, the lead molecules should ideally undergo evaluation using a comprehensive set of bioassays, rather than relying solely on the hemolysis assay during drug development. Eculizumab (Soliris, Alexion Pharmaceuticals), an anti-C5 IgG, is the first complement drug with potent activity in assays like C5 binding, and C5 cleavage inhibition along with hemolysis inhibition, and has been approved for the treatment of PNH (36). However, 18 to 35% of patients treated with eculizumab remain transfusion-dependent in the clinic due to extravascular hemolysis after C3b deposition (16), which results from the lack of evaluation of C3b deposition in the drug development stage. This clinical observation is consistent with the results of the C3b deposition assay in our study (Fig. 5). Given the absence of suitable predictive preclinical models of some complement-related diseases like GA (37), the bioassays developed in this study are particularly valuable for C3 drug discovery and optimization.

In conclusion, our study provides a comprehensive understanding of the inhibitory mechanism of C3 inhibitors, based on the SAR of C3 and complement biology. This understanding is crucial for the design of effective C3 therapeutics. We have successfully identified the correlation between the epitope, inhibition processes, and functional properties within the complement cascade. Our research suggests that the epitope selection significantly influences both the inhibition processes and potency. Targeting a specific epitope has the potential to modulate the complement system, leading to diverse biological effects that can address various clinical needs in complement-related diseases.

## Experimental procedures

### Reagents

SMM 293-TII medium (cat#M293TII) was purchased from Sinobiological. PEI (cat#78PEI4000) was purchased from Biohub International Trade Co, Ltd. HEK293-F cell line was provided by the Technology Center for Protein Research at Tsinghua University. pCDNA3.1 plasmid was provided by General Biol. Ni-NTA agarose (cat#SA004025) was purchased from Smart Life Sciences. C3 (cat#A113), C3b (cat#A114), FB (cat#A135), FD (cat#A136), C4b (cat#A108), C2 (cat#A112), C1s (cat#A104), C5 (cat#A120), C3a-desArg (cat#A119), FH (cat#A137), FI (cat#A138), Mg-EGTA (cat#B106), GVB^0^ (cat#B101), GVB^++^ (cat#B100) and zymosan (cat#B400) were purchased from Complement Technology. ELISA coating buffer (cat#R20934) and hemolysin (anti-sheep RBC antibody, cat# S25861-10) were purchased from Yuanye Bio-Technology. Non-fat milk solution (cat#LP0033B) and ELISA stop solution (cat#C1058) were purchased from Solarbio. HRP-conjugated anti-His antibody (cat#HRP-66005) was purchased from Proteintech. HRP-conjugated anti-VHH antibody (cat#A01861) was purchased from GenScript. TMB substrate solution (cat#PA107) was purchased from Tiangen. BS3 (Bis[sulfosuccinimidyl] suberate, cat#C15178241) was purchased from Macklin. 4%-20% SDS-PAGE (cat#36256ES10) and Gravity Chromatography Columns (cat#20523ES03) were purchased from Yeasen. 1M Tris-HCl pH 8.0 (cat#A01861) was purchased from Solarbio. Rabbit Red Blood Cells (rRBCs, cat#JLSW-403) and sheep Red Blood Cells (shRBCs, cat#JLSW-401) were purchased from Jiulong Biopharmaceutical. Normal human serum (NHS, cat#100-512) and rabbit serum (cat#100-116) were purchased from Gemini. Heat-aggregated IgG (cat#I4506), trypsin (cat#T8658), and chymotrypsin (cat#C6423) were purchased from Sigma. Wild-type alpha-lytic protease (cat# #33036) was purchased from Cell Signaling Technology. Biotin anti-human C5b-9 (cat#ab237699) was purchased from Abcam. HRP-labeled streptavidin (cat#D111054-0100) was purchased from Sangon Biotech. 96-well ELISA plate (cat#514201) was purchased from Nest. Anti-C3a/C3a-desArg antibody [2991] (cat#GTX54437) was purchased from GeneTex. Biotin anti-human C3a/C3a(desArg)/C3 antibody (cat#518002) and APC-labeled anti-C3b antibody (cat#846106) were purchased from BioLegend.

### Production of MIs and C345C domain

The identities of the three MIs are described in Table S1. MI1 was purchased from the VuRoyal Pharmaceutical Company LLC, which was developed and produced by the Apellis Therapeutics, (Syfovre, pegcetacoplan injection, Lot: 1980264). MI1 contains pegcetacoplan, which is a symmetrical molecule comprised of two identical pentadecapeptides covalently bound to the ends of a linear 40 kDa PEG molecule (13). The comprehensive drug information and certificate of analysis (COA) standards of Syfovre can be found in the FDA database (‘https://www.fda.gov/drugsatfda’). MI2 and MI3 are two protein inhibitors, nanofitin and nanobody, respectively. The discovery process and identity of MI2 were described in the previous study (22), and its amino acid sequence is listed in Table S3. MI3 used in this study was kindly provided by Quaerite Biopharm Research, which is a proprietary nanobody.

MI2, MI3, and the C345C domain were cloned into pCDNA3.1(+) plasmid with the addition of the signal peptide (MDAMKRGLCCVLLLCGAVFVSPS) at the N-terminal, and the linker (GGGGS) and 6xHis (HHHHHH) at the C-terminal of the protein. The sequences of plasmid and amino acid of MIs and C345C domain are listed in Tables S2 and S3. The sequences of all plasmids were verified by sequencing before expression. The results of plasmids sequencing are supplied in Supporting Information. HEK293-F cells were cultured in SMM 293-TII medium at 5% CO2 and 37 °C for 4 to 5 days before expression. The cells were then transfected with the plasmid and PEI at a final concentration of 1 μg/ml and 3 μg/ml, respectively, at a density of 1.5 to 2.0 × 10^6^ cells/ml after being sub-cultured for 3 to 4 passages.

Expressed proteins in the supernatant were purified by Ni-NTA agarose and ion exchange (GigaCap Q-650S TOSOH for MI3, GigaCap Q-650M TOSOH for MI2 and C345C domain, Tosoh Corporation, Japan). In purification by Ni-NTA agarose, 3 ml Ni-NTA agarose was added to 1 L supernatant of cell culture medium and incubated at 4 °C overnight. The Ni-NTA agarose was recovered through Gravity Chromatography Columns, and washed with 50 ml of 10 mM imidazole in PBS. After washing, the target protein was eluted from the Ni-NTA agarose with 30 ml 200 mM imidazole in PBS, and concentrated by Amicon Ultra-0.5 Centrifugal Filter 3 kDa MWCO Millipore. In cation exchange (GigaCap Q-650S TOSOH) purification, the equilibration buffer is 20 mM citric acid, pH 6.0 and the elution buffer is 20 mM citric acid, pH 6.0, 1M NaCl. In anion exchange (GigaCap Q-650M TOSOH) purification, the equilibration buffer is 25 mM Tris-HCl, pH 8.0 and the elution buffer is 25 mM Tris-HCl, pH 8.0, 1 M NaCl.

The 4%-20% SDS-PAGE and size-exclusion high-performance liquid chromatography (SEC-HPLC) were applied to analyze the purity of the protein sample (Fig. S1). The purity of all proteins in this study was greater than 95%. In 4 to 20% SDS-PAGE, the Tris-Mops-SDS running buffer, *i.e.*, 50 mM Tris, 50 mM HEPES, 0.1% SDS, 2 mM EDTA, was used. In SEC-HPLC, prominence-i9 (LC-2030, Shimadzu, Japan) high-performance liquid chromatography system, coupled with Zenix-C SEC-300 (3 μm, 7.8∗300 mm, Sepax Technologies, DE, USA) was used. The mobile phase in SEC-HPLC is 50 mM PB, 300 mM NaCl, pH 6.8. Protein concentration was measured by UV absorbance at 280 nm (Nanodrop 2000, Thermo Scientific) based on the extinction coefficient, which is calculated based on protein sequence at https://web.expasy.org/protparam/.

### Binding affinity and specificity to C3 and C3b

The binding affinity and specificity to C3 and C3b of MI3 were analyzed by ELISA. Each well in the 96-well ELISA plate was coated with 100 μl of 2 μg/ml C3 or C3b or C3a or C4b or C5 or C345C domain (recombinant expression) diluted in ELISA coating buffer and incubated overnight at 4 °C. The plate was washed with 200 μl PBST (PBS, 0.1% (v/v) Tween 20) three times and blocked for 1 h with 5% non-fat milk solution at 37 °C, followed by three washes with 200 μl PBST. A concentration gradient diluent (100 μl) of MIs diluted in PBST was added and incubated at 37 °C for 1 h. Followed by washing with PBST three times, 100 μl of the HRP-conjugated anti-His antibody diluted 1:7000 in PBST was added to each well and incubated at room temperature for 45 min. For the C345C domain (recombinant expression), 100 μl of the HRP-conjugated anti-VHH antibody diluted 1:5000 in PBST rather than anti-His antibody was added to each well. Followed by washing with PBST three times, 100 μl of TMB substrate solution was added and incubated at 37 °C for 15 min 100 μl of ELISA stop solution was added and the results were read at OD450 using a microplate reader (SpectraMax Gemini XPS/EM Microplate Readers, Molecular Devices).

### Chemical Cross-Linking mass spectrometry (CX-MS)

In CX-MS, the MI3 was incubated with 0.5 mg/ml C3b or C3 with a 2:1 M ratio in PBS at 4 °C for 1h to obtain the C3b-MI3 or C3-MI3 complex. The protein complexes were cross-linked with 12.5 mM BS3 for 30 min at room temperature. After the cross-linking reaction, 50 mM tris-HCl pH 8.0 was added to the sample to quench the reaction for 20 min at room temperature. After protein reduction and alkylation, the cross-linked samples were separated by 4%-20% SDS-PAGE. The regions corresponding to the cross-linked protein complex were cut and in-gel digested with trypsin and chymotrypsin, or trypsin and wild-type alpha-lytic protease, and the MS analysis was performed as described in (38). The results of BS3 crosslinking and MS analysis are described in Tables S4 and S5, including the identification of the crosslinked fragments from C3, C3b, and MI3.

### Molecular docking and Molecular Dynamics (MD) simulation

Molecular docking of C3b and C3 with MI3 was performed in HADDOCK2.4 (https://wenmr.science.uu.nl/haddock2.4/ (39)) with the restriction from CX-MS and the binding of C345C domain. The structure of C3b and C3 were obtained from the RCSB Protein Data Bank (PDB: 7BAG, PDB: 2A73). The structure of MI3 was predicted by AlphaFold3 (40), and the predicted structures were supplied in Supporting Information. The details of restrictions in HADDOCK2.4 are described in Table S6, and all the docking results shown in Fig. S4 were supplied in Supporting Information. MD simulation (41) was applied to simulate the *in silico* stability of the C3b-MI3 and C3-MI3 complex structure from HADDOCK2.4. MD simulations were performed using GROMACS 2020.05 software as described previously (42). Briefly, the system was prepared by placing protein complex structures in a periodic cubic box with a boundary size of 1.2 nm. Sodium chloride was added to achieve a concentration of 0.15 mol/L for charge neutralization. A maximum descent algorithm was utilized for energy minimization, with up to 5000 steps employed to optimize the system structure. Pre-equilibration phases, including 1 ns of NVT and 1 ns of NPT equilibration, were performed. The system temperature was maintained at 310 K, and the pressure was set to 1 bar using the V-rescale algorithm and the Parrinello-Rahman algorithm. The duration of the formal simulation is 100 ns with a time step of 2 fs. The CHARMM36 force field was employed for all simulations. The coordinates of the protein complex structures generated during the simulation, including the first, last, and central structures shown in Fig. S5, were supplied in Supporting Information. The Root Mean Square Deviation (RMSD) values of the corresponding protein groups during the simulation were calculated according to Equation 1, including the MI3, the C3 or C3b, and the complex of MI3 with C3 or C3b. The calculation of the three groups of RMSD was performed in the “gmx rms” module of GROMACS 2020.05 software.

(1) $$R\text{MSD}\;\left(t_{1},t_{2}\right)={\left[\frac{1}{\sum_{i=1}^{N}m_{i}}\sum_{i=1}^{N}m_{i}{\left\|r_{i}\left(t_{1}\right)-r_{i}\left(t_{2}\right)\right\|}^{2}\right]}^{1/2}$$

where *N* is the number of atoms, *m*_*i*_ is atomic weight, and *r*_*i*_*(t*_*1*_*)* is the space coordinate vector of the λh atom at time = *t*_*1*_. *r*_*i*_*(t*_*2*_*)* is the coordinate vector of the atom in the reference structure used in the least-squares fitting method. Here, the first frame (0 ns) of the simulation was defined as the reference structure, and its atomic coordinate vector is *r*_*i*_*(t*_*0*_*)*.

### Preparation of C3b-MI3 complex

2-fold molar excess of MI3 was added to C3b and incubated for 30 min at 4 °C. The complex was purified by a superdex 200 increase column (GE Healthcare) equilibrated in 20 mM Tris-HCl (pH 8.0) and 150 mM NaCl. The early fraction corresponding to the C3b-MI3 complex detected by SDS-PAGE was concentrated with ultrafiltration tubes (3 kD).

### Cryogenic Electron Microscopy (cryo-EM)

For grid preparation, sample vitrification was performed using an FEI Vitrobot at 10 °C and 100% humidity. 3.5 μl C3b-MI3 complex (0.8 mg/ml) was applied to 300 mesh glow-discharged gold grid (Quantifoil R 1.2/1.3) coated with holey carbon film. Excess liquid was removed by blotting with a blot force of one and blot time of 2 s before the grid was plunge-frozen in liquid ethane. For data collection, cryo-EM data were captured using a 200 kV FEI Tecnai Arctica transmission electron microscope (FEI). Images were recorded with a K2 direct electron detector (Gatan) operating in super-resolution mode. The dataset was collected using SerialEM software (43), with a pixel size of 0.836 Å and an accumulated dose of 50 e−/Å^2^. For cryo-EM data processing, the dataset was subjected to beam-induced motion correction using MotionCor2 (44) and processed in cryoSPARC (45). Multiple rounds of 2D classification were performed to remove junk particles (46). An initial 3D model was generated through *ab initio* reconstruction. The map was progressively improved by non-uniform refinements iteratively. Structural figures were generated using PyMOL (Schrödinger, Inc.) and UCSF Chimera (47).

### Hemolysis assay

In the AP-mediated hemolytic assay, GVB-MgEGTA was used to prepare the sample and wash the rRBCs. GVB-MgEGTA was prepared by adding Mg-EGTA (0.1 M) to GVB^0^ at 1:100 (v/v). The final concentration of MgEGTA in the GVB-MgEGTA is 1 mM. rRBCs were washed three times and resuspended with twice the volume of GVB-MgEGTA before use. NHS was activated by incubating at 37 °C for 30 min before use. A concentration gradient diluent (75 μl) of the MIs was incubated with 75 μl activated NHS and 100 μl rRBCs at 37 °C for 30 min. Maximum lysis control was established by incubating 75 μl GVB-MgEGTA with 75 μl activated NHS and 100 μl rRBCs at 37 °C for 30 min, and minimum lysis control was established by incubating 150 μl GVB-MgEGTA and 100 μl rRBCs at 37 °C for 30 min. After incubation, 70 μl 250 mM EDTA was added to stop the reaction followed by centrifugation. The release of hemoglobin was measured at OD412 using a microplate reader (SpectraMax Gemini XPS/EM Microplate Readers, Molecular Devices). The percentage of lysis was calculated with the following formula: % lysis = ([Maximum lysis control - Sample OD412]/[Maximum lysis control – minimum lysis control]) × 100.

In the CP-mediated hemolytic assay, shRBCs were washed three times with GVB^++^. shRBCs were then sensitized by mixing at 1:1 (v/v) with hemolysin that is diluted 1:2000 in GVB^++^, and incubating at 37 °C for 30 min. A concentration gradient diluent (75 μl) of the MIs was incubated with 25 μl activated NHS and 100 μl sensitized shRBCs at 37 °C for 1 h. Maximum lysis control was established by incubating 75 μl GVB^++^ with 25 μl activated NHS and 100 μl sensitized shRBCs at 37 °C for 1 h and minimum lysis control was established by incubating 75 μl GVB^++^ with 25 μl GVB^++^ and 100 μl sensitized shRBCs at 37 °C for 1 h. 70 μl 250 mM EDTA was added to stop the reaction followed by centrifugation. The release of hemoglobin was measured at OD412 using a microplate reader (SpectraMax Gemini XPS/EM Microplate Readers, Molecular Devices). The percentage of lysis was calculated as AP hemolysis.

### C5 cleavage assay

In the C5 cleavage assay, each well in the 96-well ELISA plate was coated with 100 μl of 20 μg/ml zymosan or 15 μg/ml heat-aggregated IgG diluted in ELISA coating buffer and incubated overnight at 4 °C. The plate was washed with 200 μl PBST in AP or D-PBST (D-PBS, 0.1% (v/v) Tween 20) in CP three times, and blocked for 1 h with 5% non-fat milk solution at 37 °C, followed by three washes with 200 μl PBST (AP) or D-PBST (CP). Next, 100 μl 15% NHS in AP or 5% NHS in CP supplemented with MIs at the indicated concentration diluted in GVB-MgEGTA or GVB^++^ was added to each well, and incubated at 37 °C for 1.5 h. After washing with PBST three times, 100 μl of biotin anti-human C5b-9 diluted 1:1000 in PBST was added to each well and incubated at 37 °C for 1 h. Followed by three washed with PBST, 100 μl of HRP-labeled streptavidin diluted 1:2000 in PBST was added to each well and incubated at room temperature for 45 min. Followed by three washed with PBST, 100 μl of TMB substrate solution was added and incubated at 37 °C for 15 min 100 μl of ELISA stop solution was added and the results were read at OD450 using a microplate reader (SpectraMax Gemini XPS/EM Microplate Readers, Molecular Devices).

### C3 convertase formation assays and C3a release detection

In the C3 cleavage assay, plasma concentrations were used as the reference to set the concentration of each purified complement component. The concentration of each complement component decreased proportionally according to the plasma concentration based on the response value in C3a-desArg quantification by the ELISA experiment described below. The response value (OD450) of the ELISA experiment is between 0 to 2. The GVB-MgEGTA or GVB^++^ was used to prepare samples in the AP or CP respectively. In AP-mediated C3 cleavage, 29 nM FB was incubated with a concentration gradient diluent of the MIs. 20 nM C3b, 3.85 nM FD, and 100 nM C3 were then added and incubated for 45 min at room temperature. Since FD is the rate-limiting enzyme in the assembly of C3bBb, to assemble the active C3bBb *in vitro*, the concentration of FD was increased from 1.28 nM (calculated by the plasma concentrations of FD) to 3.85 nM. In CP-mediated C3 cleavage, 2.25 nM C2 was incubated with a concentration gradient diluent of the MIs. 24 nM C4b, 2.75 nM C1s, and 100 nM C3 were then added and incubated for 45 min at room temperature. The C3a was converted to C3a-desArg by adding rabbit serum (diluted in 1:20 (v/v)) at room temperature for 15 min. The reactions were quenched by 0.1 M EDTA, and the C3a-desArg was quantified.

In the C3 convertase formation assay, two incubation methods were applied, including “C3 incubation” and “C3b incubation”. The GVB-MgEGTA was used to prepare samples. In “C3 incubation”, 50 nM C3 was incubated with a concentration gradient diluent of the MIs. The C3 convertase C3bBb was prepared by incubating 100 nM C3b, 100 nM FB, and 1 nM FD for 10 min at room temperature, and subsequently added to the C3-MIs complex with 30 min incubation at room temperature. The C3a was converted to C3a-desArg by rabbit serum (diluted in 1:20 (v/v)) at room temperature for 15 min. The reactions were quenched by adding 0.1 M EDTA, and the C3a-desArg was quantified. In “C3b incubation”, 100 nM C3b was incubated with a concentration gradient diluent of the MIs, followed by adding 100 nM FB, and 1 nM FD. 100 nM C3 was added to each sample and incubated for 30 min at room temperature. The C3a was converted to C3a-desArg by adding rabbit serum (diluted in 1:20 (v/v)) at room temperature for 15 min. The reactions were quenched by adding 0.1 M EDTA, and the C3a-desArg was quantified.

For C3a-desArg quantification, each well in the 96-well ELISA plate was coated with 100 μl of 1 μg/ml anti-C3a/C3a-desArg antibody [2991] diluted in ELISA coating buffer and incubated overnight at 4 °C. The plate was washed with 200 μl PBST three times and blocked for 1 h with 5% non-fat milk solution at 37 °C, followed by three washes with 200 μl PBST. The sample (100 μl) was added and incubated at 37 °C for 1 h. Followed by washing with PBST three times, 100 μl of biotin anti-human C3a/C3a(desArg)/C3 antibody diluted 1:1000 in PBST was added and incubated at 37 °C for 1 h. Followed by washing with PBST three times, 100 μl of HRP-labeled streptavidin diluted 1:2000 in PBST was added and incubated at room temperature for 45 min. Followed by washing with PBST three times, 100 μl of TMB substrate solution was added and incubated at 37 °C for 15 min 100 μl of ELISA stop solution was added and the results were read at OD450 using a microplate reader (SpectraMax Gemini XPS/EM Microplate Readers, Molecular Devices, USA).

### C3b deposition assay

After AP-mediated hemolysis under 5 μM MIs, rRBCs were collected and washed with PBS. rRBCs were incubated on ice with APC-labeled anti-C3b antibody diluted 1:20 in 3% BSA for 30 min in the dark. rRBCs were then washed three times and analyzed by FACScan (BD Biosciences). At least 10,000 events in each sample were acquired, and analyzed by FlowJo (version 7.6.5 software; Tree Star).

### FB cleavage assay and FB competitive binding assay

In FB cleavage assay, 2 μM C3b diluted in GVB-MgEGTA was incubated with 20 μM MIs at room temperature for 30 min 2 μM FB and 0.5 μM FD were then added. The reaction mixture was incubated at room temperature, and samples for 4%-20% SDS-PAGE analysis were taken after 15 min with non-reducing SDS-PAGE loading dye.

In FB competitive binding assay, each well in the 96-well ELISA plate was coated with 100 μl of 1 μg/ml C3b diluted in ELISA coating buffer and incubated overnight at 4 °C. The plate was washed with 200 μl PBST three times and blocked for 1 h with 5% non-fat milk solution at 37 °C, followed by three washes with PBST. The MIs were diluted to 1 nM in PBST containing 0.01 M MgCl_2_, and the concentration gradient diluent of the FB with equal volume was added to the MIs solution. The mixture of MIs and FB was added and incubated at 37 °C for 1h. Followed by three washes with PBST, 100 μl of HRP-conjugated anti-His antibody diluted 1:7000 in PBST was added and incubated at room temperature for 45 min. Followed by three washes with PBST, 100 μl of TMB substrate solution was added and incubated at 37 °C for 15 min 100 μl of ELISA stop solution was added and the results were read at OD450 using a microplate reader (SpectraMax Gemini XPS/EM Microplate Readers, Molecular Devices).

### FH-mediated FI degradation assay

FH-mediated FI degradation assay was performed in GVB-MgEGTA. 2 μM C3b was incubated with 10 μM MIs at room temperature for 30 min 0.2 μM FH and 0.2 μM FI were then added. The reaction mixture was incubated at 37 °C, and samples for 4%–20% SDS-PAGE analysis were taken after 2 h. The samples were mixed with reducing SDS-PAGE loading dye and boiled at 95 °C for 30 s.

## Data availability

All data are contained within the article.

## Supporting information

This article contains supporting information (48).

## Conflict of interest

The authors declare that they have no conflicts of interest with the contents of this article.

## Supporting information

C3b-C345C_gene_sequencing

C3b-MI3_central

C3b-MI3_first

C3b-MI3_last

C3-MI3_first

C3-MI3_last

Figure_S4A

Figure_S4B

Figure_S4C

Figure_S4D

Figure_S4E

Figure_S4F

Figure_S4G

MI2_gene_sequencing

MI3.pdb

MI3_gene_sequencing
