# Supplementary material for: Modulating the complement system through epitope-specific inhibition by complement C3 inhibitors
Source: J Biol Chem. 2025 Jan 31;301(3):108250. doi: 10.1016/j.jbc.2025.108250 (PMC11910092; doi:10.1016/j.jbc.2025.108250)
Supplement: C3b-C345C_gene_sequencing [file mmc1.zip › Supporting_Information/Supporting_Information.docx]

**Supporting Information

Modulating the complement system through epitope-specific inhibition by complement C3 inhibitors**

Zhidong Chen^1†^, Mingshuang Wang^1,2†^, Wenqian Duan^1^, Yi Xia^1^, Huiqin Liu^2*^, Feng Qian^1*^

1 School of Pharmaceutical Sciences, Beijing Frontier Research Center for Biological Structure, and Key Laboratory of Bioorganic Phosphorus Chemistry & Chemical Biology (Ministry of Education), Tsinghua University, Beijing, P. R. China

2 Quaerite Biopharm Research Co., Ltd., Beijing, PR China

^†^ These authors contributed equally to this work

* **For correspondence**: Huiqin Liu, liu.huiqin@quaerite.com; Feng Qian, qianfeng@tsinghua.edu.cn.

This manuscript contains eight supporting figures, six supporting tables, three gene

sequences, and fourteen protein structure files.

**Figure S1.** The purity of MI1, MI2, MI3, and C345C domain verified by SDS-PAGE and SEC-HPLC.

**Figure S2.** Binding specificity and affinity of MI3 to C3 and C3b detected by ELISA.

**Figure S3.** The crosslinked residues detected by CX-MS in (A) C3-MI3 and (B) C3b-MI3.

**Figure S4.** The results of molecular docking restricted by all possible restrictions from CX-MS.

**Figure S5.** MD simulation of the predicted C3b-MI3 and C3-MI3 complex structures.

**Figure S6.** The low-resolution structure of the C3b-MI3 complex solved by cryo-EM.

**Figure S7**. Structural basis of the inhibition mechanism and effects of MIs.

**Figure S8**. Targeting C345C domain inhibits FH-mediated FI degradation of C3b (endogenous C3b regulation by FI degradation).

**Table S1.** The identities of three MIs.

**Table S2.** The plasmid sequence of MI2, MI3, and C345C domain.

**Table S3.** The amino acid sequence of MI2, MI3, and C345C domain.

**Table S4.** The crosslinking information of the C3-MI3 complex.

**Table S5.** The crosslinking information of the C3b-MI3 complex.

**Table S6.** The restrictions in molecular docking of the C3-MI3 complex and the C3b-MI3 complex


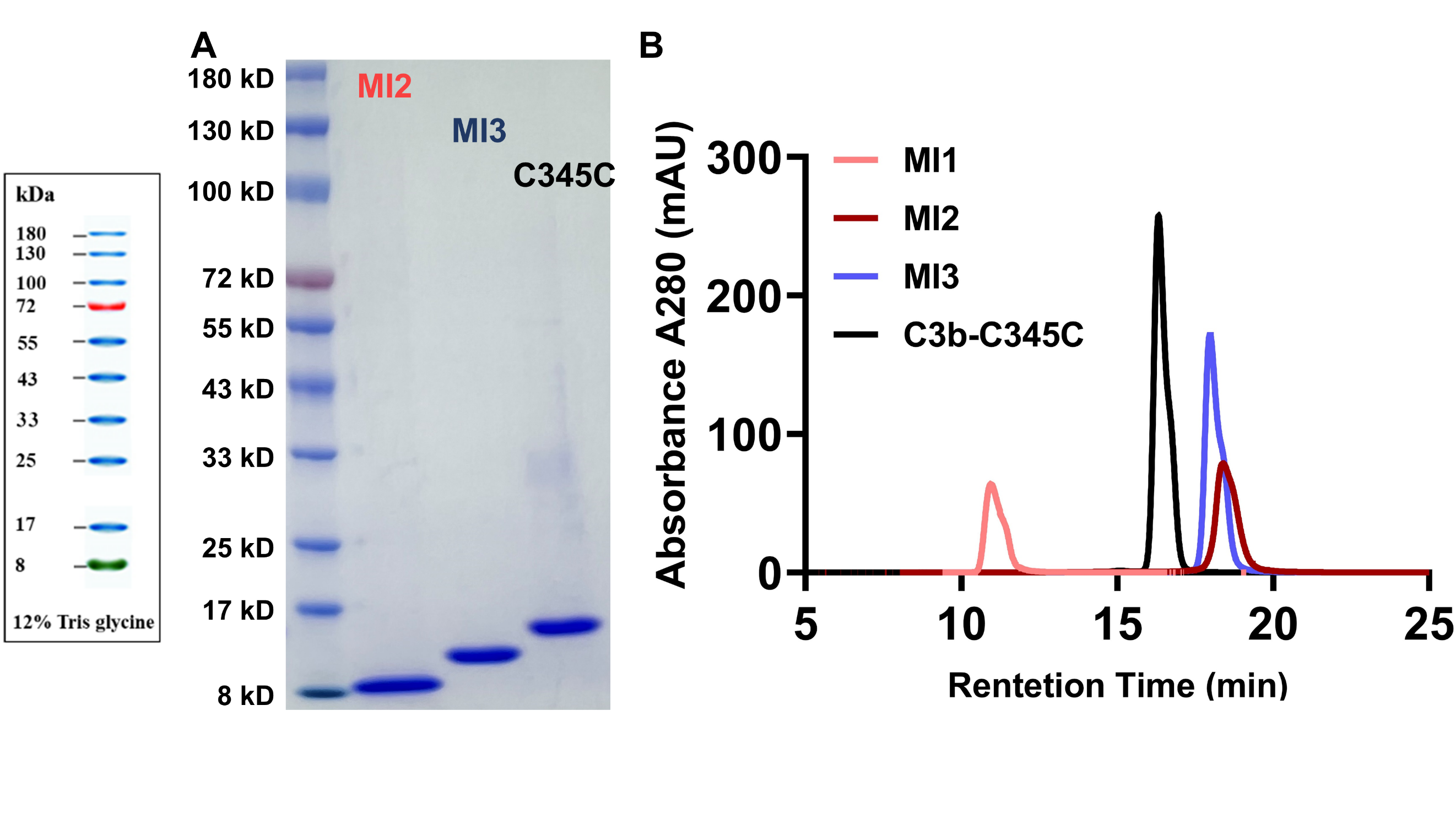


**Figure S1.** The purity of MI1, MI2, MI3, and C345C domain verified by SDS-PAGE and SEC-HPLC.


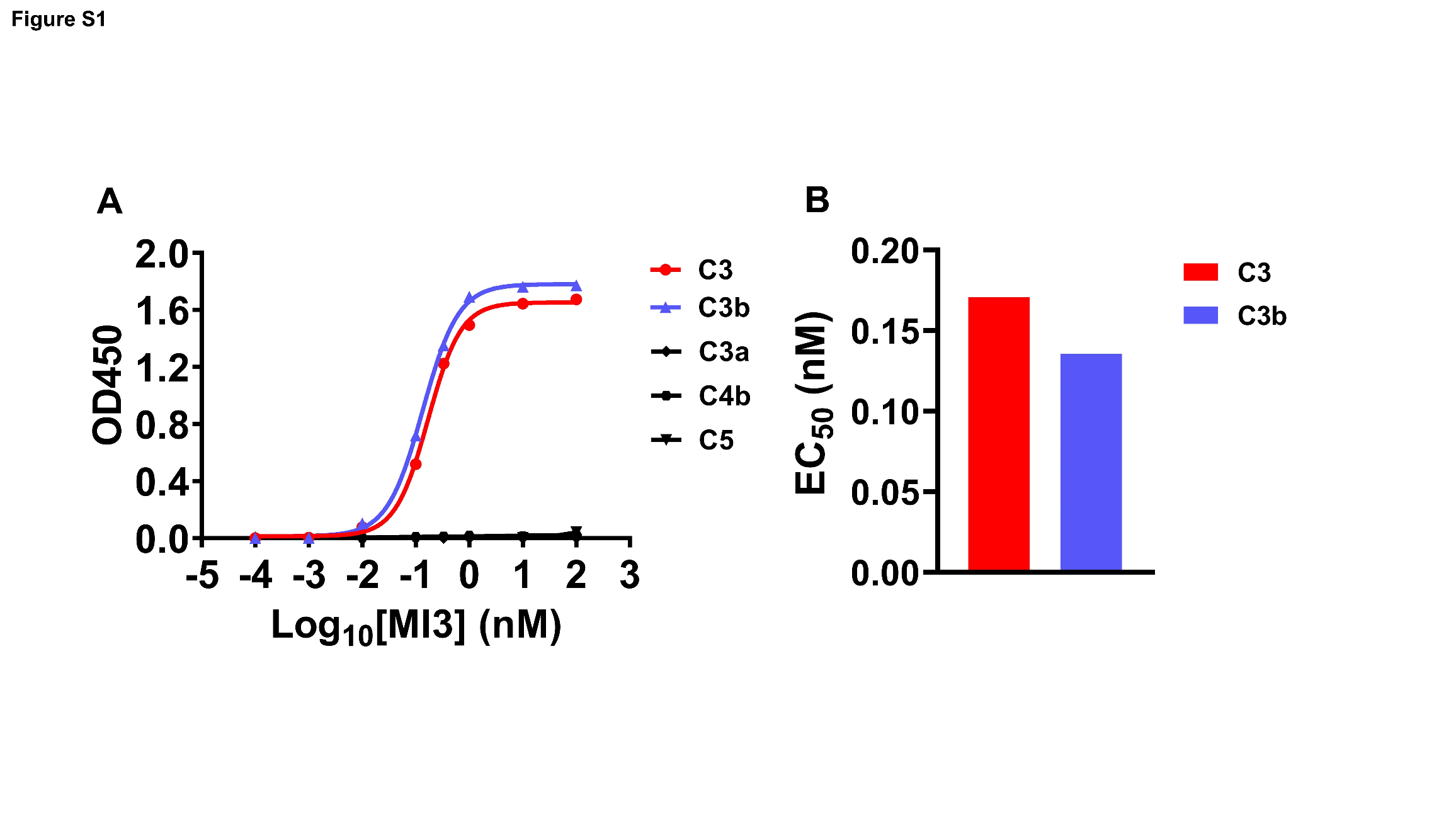


**Figure S2.** Binding specificity and affinity of MI3 to C3 and C3b detected by ELISA.


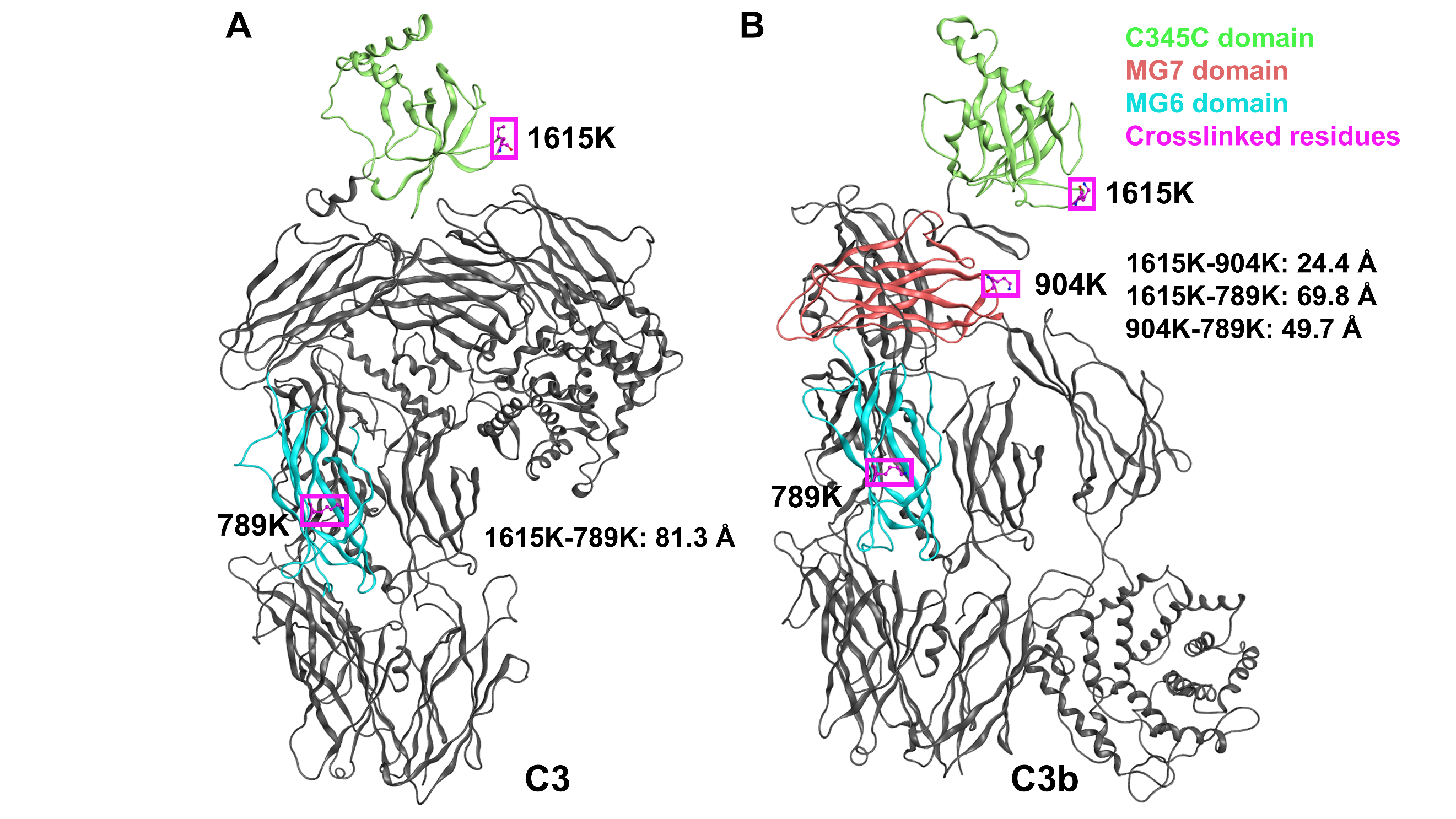


**Figure S3.** The crosslinked residues detected by CX-MS in (A) C3-MI3 and (B) C3b-MI3.


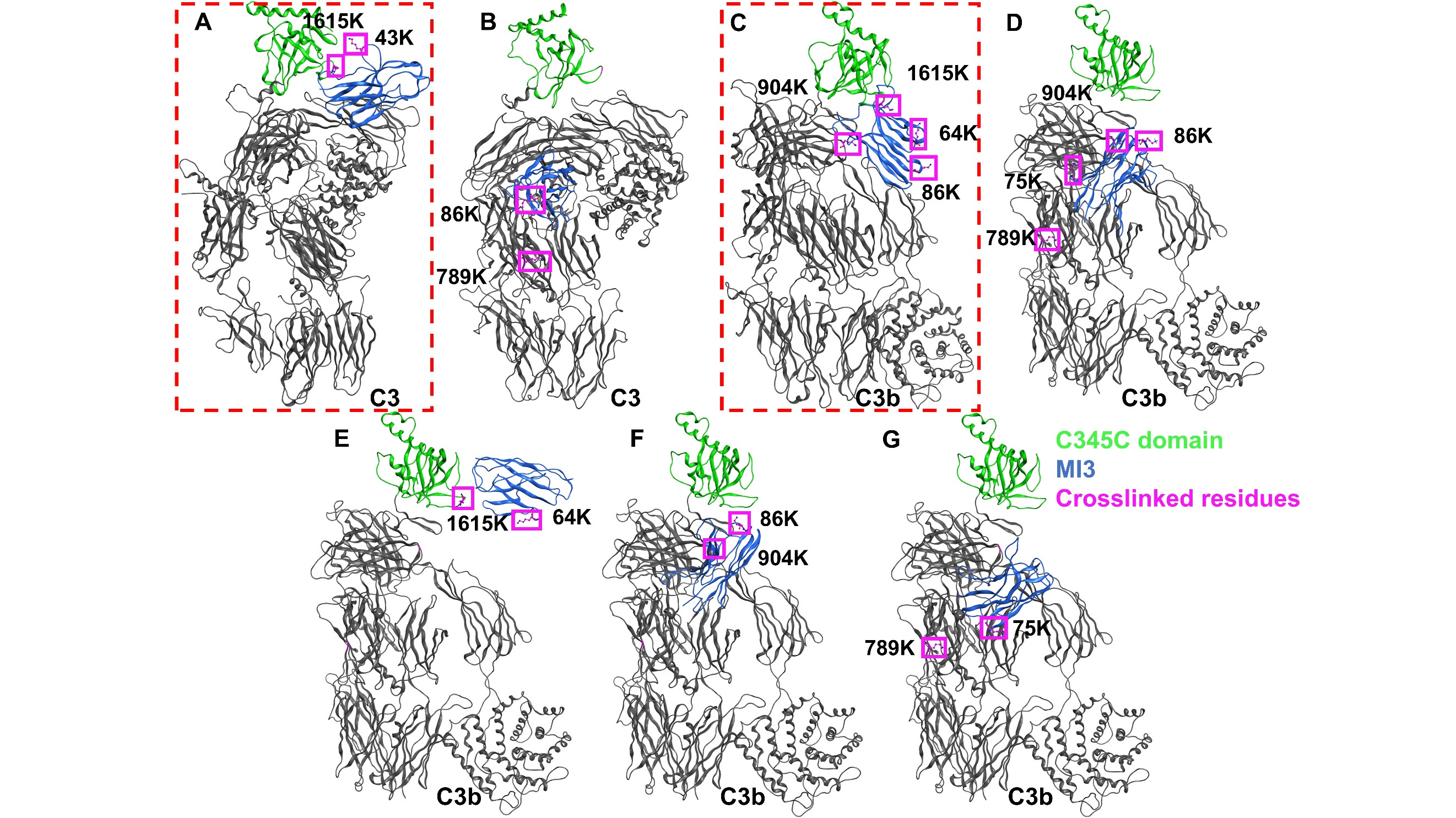


**Figure S4.** The results of molecular docking restricted by all possible restrictions from CX-MS. The pairs of crosslinked residues used for restriction in docking are highlighted by the solid box in the complex structures. The selected docking results for the MD simulation are highlighted by the dashed box. Black: C3 or C3b; Green: C345C domain of C3 or C3b; Blue: MI3.


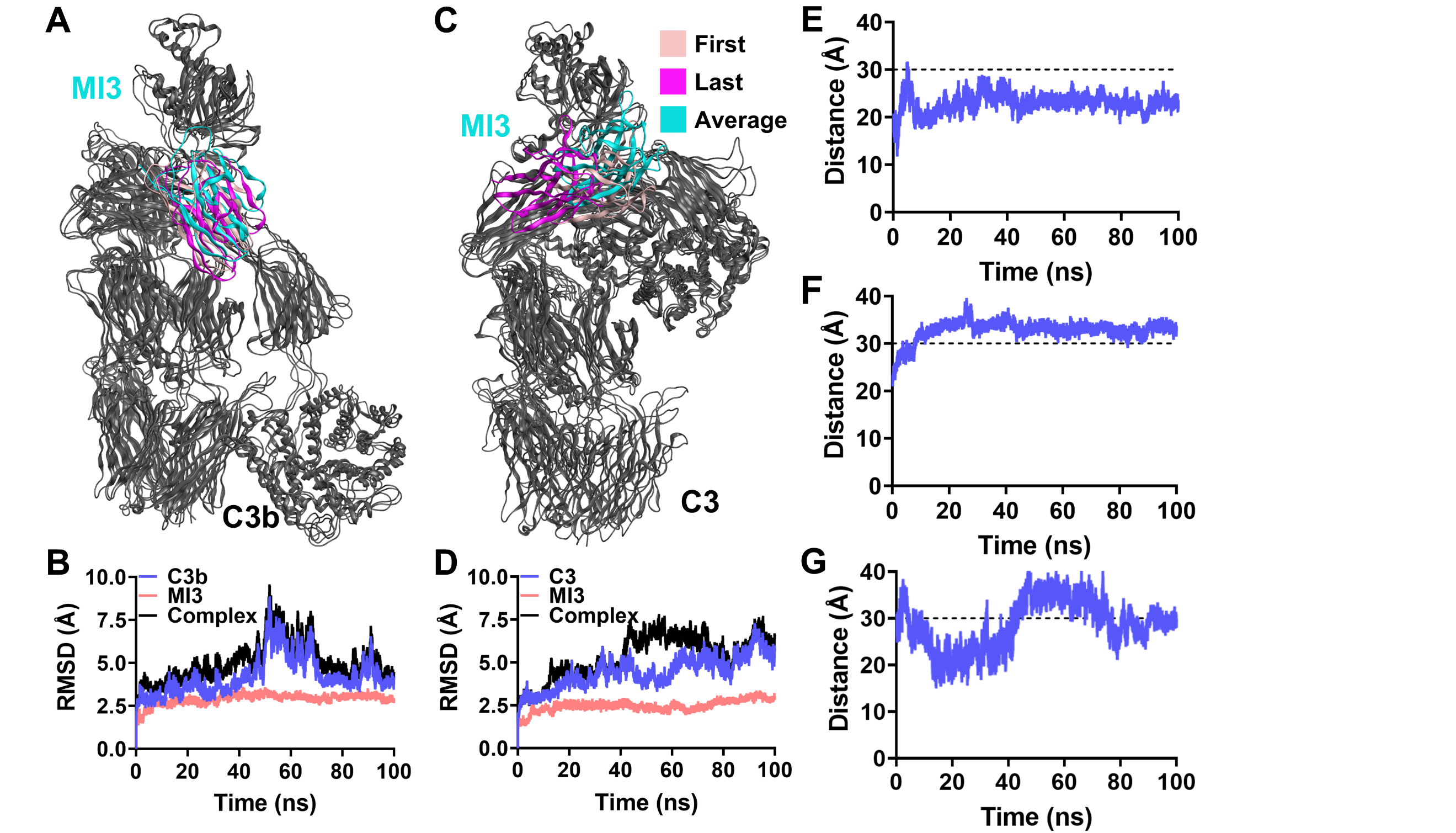


**Figure S5**. MD simulation of the predicted C3b-MI3 and C3-MI3 complex structures. (A, C) The first, last, and average conformations of the C3b-MI3 and C3-MI3 complex structures during the MD simulation. (B, D) The RMSD during the MD simulation of the C3b-MI3 complex and C3-MI3 complex. The distances between (E) C3b_K1615 and MI3_K64, (F) C3b_K904 and MI3_K86, and (G) C3_K1615 and MI3_K43 during MD simulation.


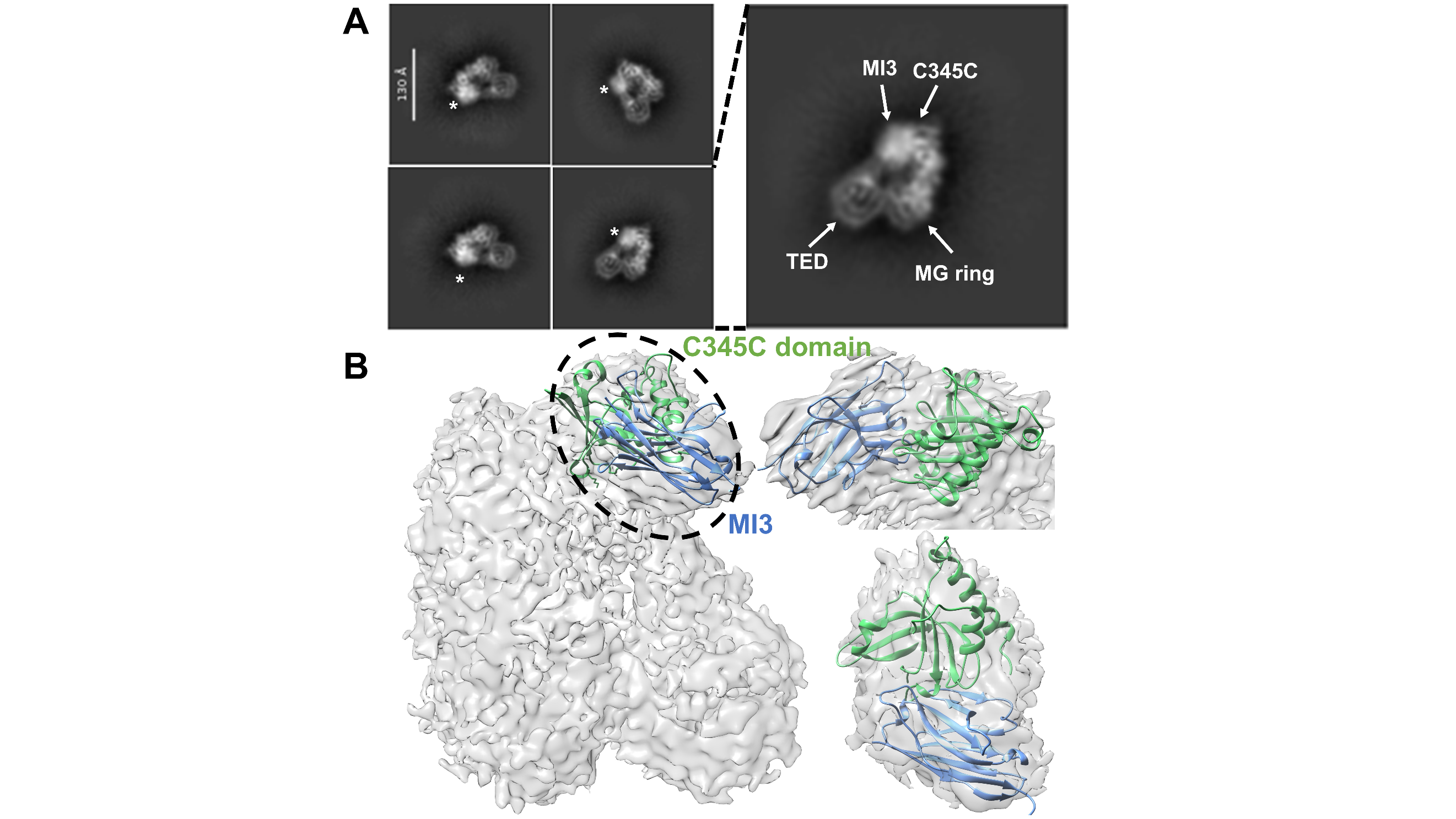


**Figure S6.** The low-resolution structure of the C3b-MI3 complex solved by cryo-EM. (A) 2D classes of the C3b-MI3 particles. Asterisk (*) marks the density of C345C and MI3; (B) The cryo-EM map of C3b-MI3 complex. The structure of C345C domain (Green) derived from PDB entry 5FO7, and the MI3 structure predicted by Alphafold3 (Blue) are fitted into the envelope (Grey).


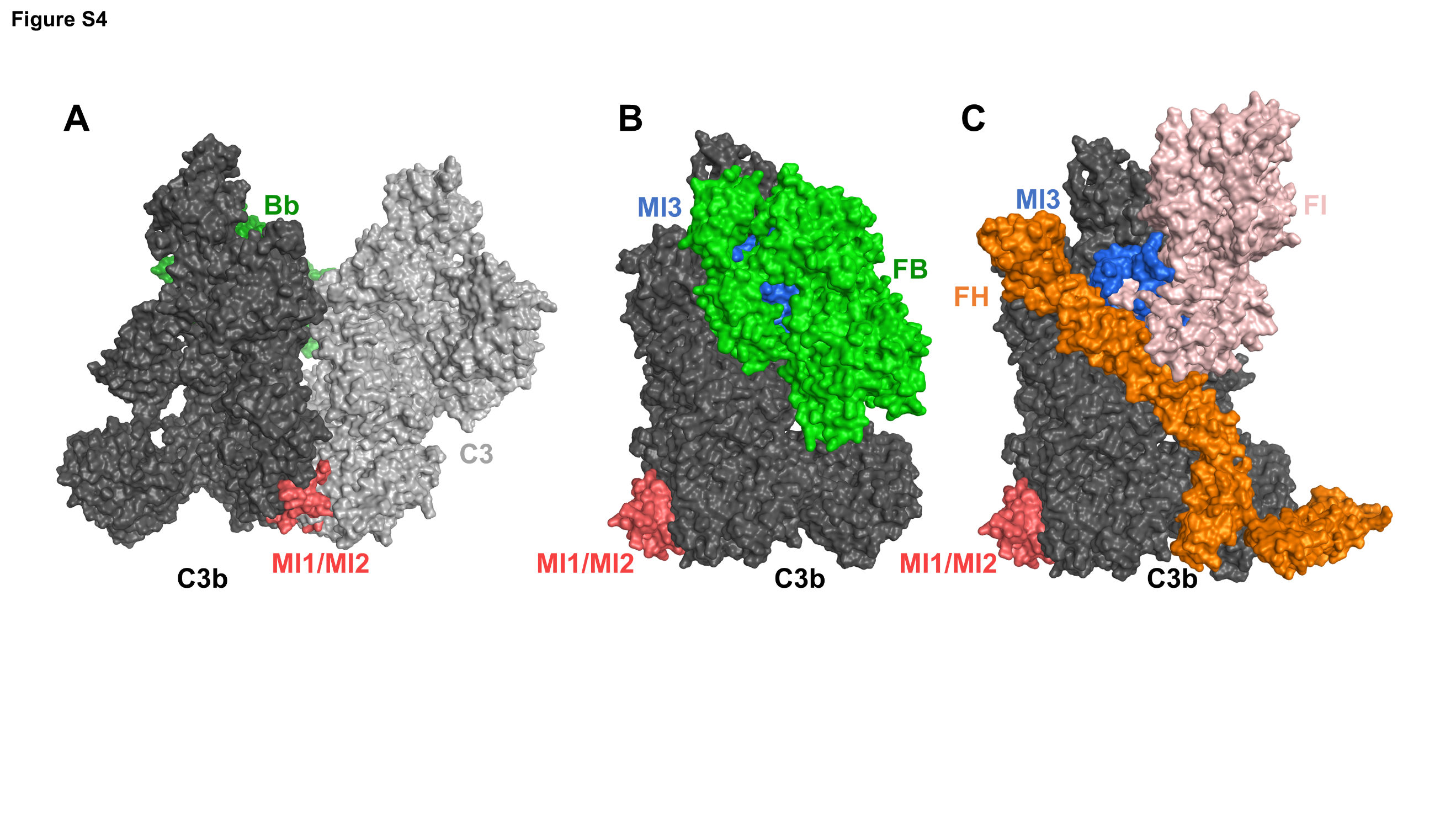


**Figure S7.** Structural basis of the inhibition mechanism and effects of MIs. (A) Model of the interaction between C3 (grey, PDB: 2A73) and C3 convertase (C3bBb) (C3b: black, Bb: green, PDB: 2WIN). MI1/MI2 (red, PDB: 7BAG and 7TV9) bind the MG4/MG5 domains of C3 and C3b, which is located at the interface of the C3-C3bBb interaction; (B) Model of the AP C3 proconvertase C3bB (C3b: black, FB: green, PDB: 2XWJ). The binding epitope of MI3 is overlaid with that of FB; (C) Model of the C3b:FI:FH complex (C3b: black, FH: orange, FI: pink, PDB: 5O32). The binding epitope of MI3 is partially overlaid with that of FH.


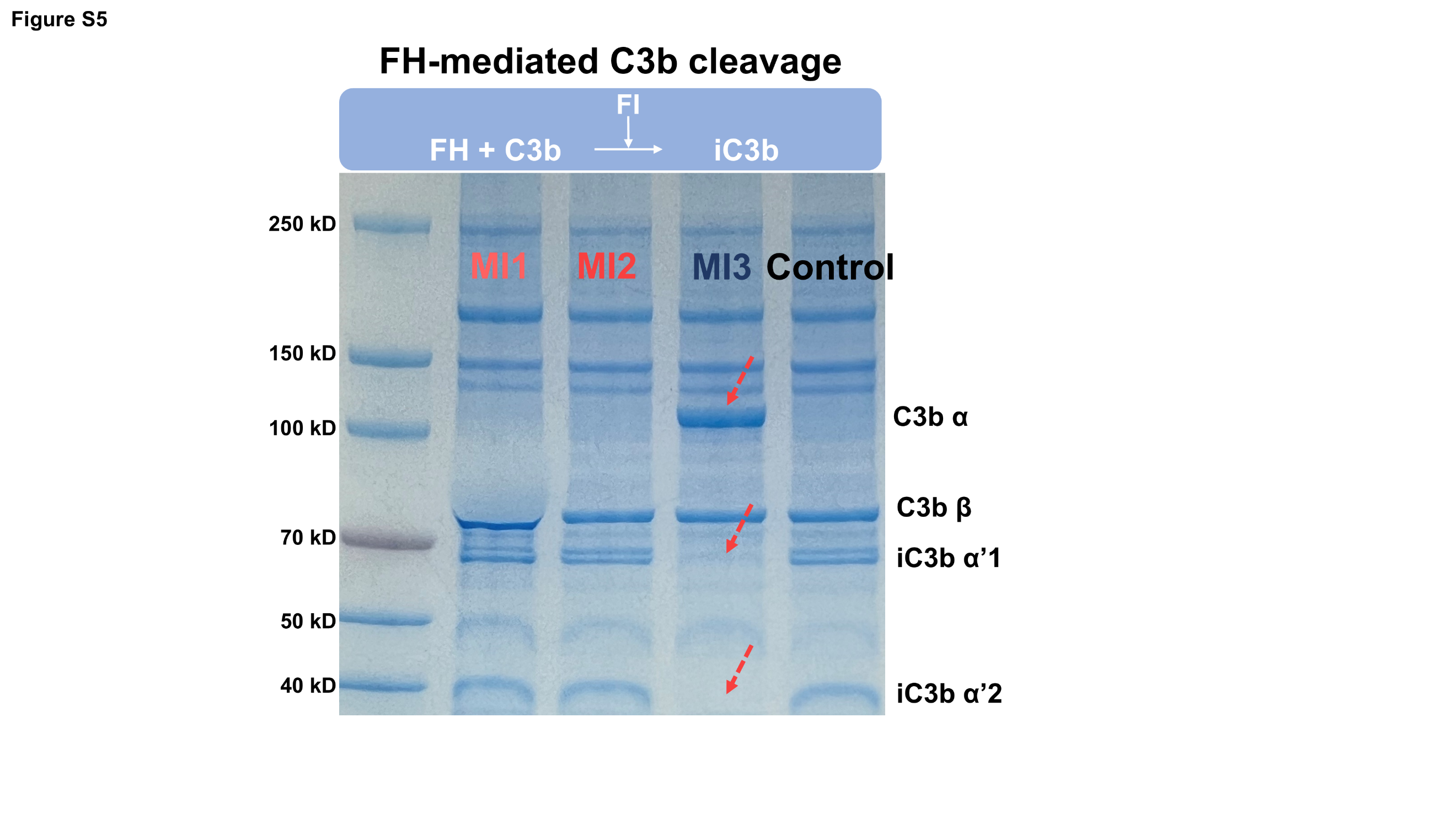


**Figure S8**. Targeting C345C domain inhibits FH-mediated FI degradation of C3b (endogenous C3b regulation by FI degradation). 2 µM C3b was incubated with 10 µM MIs at room temperature for 30 min, and 0.2 µM FH/FI were then added with 2h incubation at 37 °C. In the “control” group, buffer (GVB-MgEGTA) rather than MIs was incubated C3b and FH/FI.

**Table S1.** The identities of three MIs.

| **Model Inhibitor** | **MI1** | **MI2** | **MI3** |
| --- | --- | --- | --- |
| **Molecular Weight (kDa)** | 43.5 | 8.4 | 14.6 |
| **Identity** | PEGylation cyclic peptide | Nanofitin | Nanobody |
| **Purity** | >95% | >95% | >95% |

**Table S2.** The plasmid sequence of MI2, MI3, and C345C domain.

| **Protein** | **Plasmid sequence** |
| --- | --- |
| **MI2** | GCTAGCCACCATGGATGCCATGAAGAGAGGGCTCTGCTGTGTGCTGCTGCTGTGTGGAGCAGTGTTTGTCTCTCCCAGCGTCAAGGTGAAGTTCGACGCTACAGGCGAGGAAAAAGAGGTGGAAACCTCTAAGATCAGCGCCGTGTACAGAACCGGCAAAGATGTGCTGTTCAGCTACGACGACCAGGGCAAGATCGGATGGGGCTACGTGTCCGAGAAGGATGCCCCTAAGGAGCTGCTGGACCTGCTGGCCAGAGCCGAGCGGGAAAAGGGAGGAGGCGGCTCTCACCATCACCATCATCACTGACTCGAG |
| **MI3** | GATGTGCAGCTGCAGGAGTCTGGGGGAGGCTTGGTGCAGCCTGGGGGGTCTCTGAGACTCTCCTGTGTAGCCTCTGGAAGCACCTTCAGTACCTATCACATGGGCTGGTACCGCCAGGCTCCAGGGAAGCAGCGTGAGTTGATCGCAGGTATTACTAGTGGTGGGAGGATATCCTATGTCGACTCCGTGAAGGGCCGATTCAACATCTCCAGAGACAACGCCAAGAACACGGTGTATCTGCAAATGAACACCCTGAAACCTGAGGACACGGCCGTCTATTACTGTAACATGCTAGGAGAATTCGCAGGTCGTCCCCCCTGGGGCCAGGGGACCCAGGTCACCGTCTCC |
| **C345C domain** | GCTAGCCACCATGGATGCCATGAAGAGAGGGCTCTGCTGTGTGCTGCTGCTGTGTGGAGCAGTGTTTGTCTCTCCCAGCGCTGAAGAGAACTGCTTCATTCAGAAGTCTGATGATAAGGTGACACTGGAAGAAAGACTGGACAAGGCCTGTGAACCCGGCGTGGACTACGTGTACAAGACCAGACTCGTGAAGGTTCAGCTGTCCAACGACTTCGACGAGTACATCATGGCCATCGAGCAGACCATCAAAAGCGGCTCTGATGAGGTGCAAGTGGGCCAGCAGCGGACCTTCATCAGCCCAATCAAGTGCAGAGAGGCCCTGAAGCTGGAAGAGAAAAAGCACTACCTGATGTGGGGCCTGAGCAGCGACTTTTGGGGAGAGAAGCCTAACCTGAGCTATATCATCGGCAAGGACACATGGGTCGAGCACTGGCCTGAGGAAGATGAGTGCCAAGACGAGGAAAACCAGAAACAGTGCCAGGACCTGGGCGCCTTCACCGAGAGCATGGTGGTGTTCGGATGTCCTAATGGAGGAGGCGGCTCTCACCATCACCATCATCACTGACTCGAG |

**Table S3.** The amino acid sequence of MI2, MI3, and C345C domain.

| **Protein** | **Amino acid sequence** |
| --- | --- |
| **MI2** | VKVKFDATGEEKEVETSKISAVYRTGKDVLFSYDDQGKIGWGYVSEKDAPKELLDLLARAEREKGGGGSHHHHHH |
| **MI3** | DVQLQESGGGLVQPGGSLRLSCVASGSTFSTYHMGWYRQAPGKQRELIAGITSGGRISYVDSVKGRFNISRDNAKNTVYLQMNTLKPEDTAVYYCNMLGEFAGRPPWGQGTQVTVSSGGGGHHHHHH |
| **C345C domain** | AEENCFIQKSDDKVTLEERLDKACEPGVDYVYKTRLVKVQLSNDFDEYIMAIEQTIKSGSDEVQVGQQRTFISPIKCREALKLEEKKHYLMWGLSSDFWGEKPNLSYIIGKDTWVEHWPEEDECQDEENQKQCQDLGAFTESMVVFGCPNGGGGSHHHHHH |

**Table S4.** The crosslinking information of the C3-MI3 complex. The “Refined_Score” is generated by “plink” software developed by Mengqiu Dong’s group (1), which is a software for data analysis of cross-linked proteins coupled with mass-spectrometry analysis. The high value of “Refined_Score” (>30) represents the high confidence of the identified crosslinking peptides (1).

| **Crosslinking Peptide(site)** | **Crosslinking Protein(site)** | **Refined Score** |
| --- | --- | --- |
| WGEKPNLSY(4)-RQAPGKQR(6) | C3(1615)-MI3(43) | 30.05 |
| ISTKLM(4)-LKPEDTAVY(2) | C3(789)-MI3(86) | 34.64 |

**Table S5.** The crosslinking information of the C3b-MI3 complex. The “Refined_Score” is generated by “plink” software developed by Mengqiu Dong’s group (1), which is a software for data analysis of cross-linked proteins coupled with mass-spectrometry analysis. The high value of “Refined_Score” (>30) represents the high confidence of the identified crosslinking peptides (1).

| **Crosslinking Peptide(site)** | **Crosslinking Protein(site)** | **Refined Score** |
| --- | --- | --- |
| SDFWGEKPNLS(7)-VKGR(2) | C3b(1615)-MI3(64) | 49.46 |
| IVPLKT(5)-KPEDT(1) | C3b(904)-MI3(86) | 49.88 |
| KLMNIFLKDSITT(1)-KNTV(1) | C3b(789)-MI3(75) | 54.97 |

**Table S6.** The restrictions in molecular docking of the C3-MI3 complex and the C3b-MI3 complex.

| **Docking** | **Restrictions** |
| --- | --- |
| **C3-MI3** | assign (segid A and resid 1615) (segid B and resid 43) 30 15 0  assign (segid A and resid 789) (segid B and resid 86) 30 15 0 |
| **C3b-MI3** | assign (segid A and resid 1615) (segid B and resid 64) 30 15 0  assign (segid A and resid 904) (segid B and resid 86) 30 15 0  assign (segid A and resid 789) (segid B and resid 75) 30 15 0 |

**Reference**

1. Chen, Z.-L., Meng, J.-M., Cao, Y., Yin, J.-L., Fang, R.-Q., Fan, S.-B. *et al.* (2019) A high-speed search engine pLink 2 with systematic evaluation for proteome-scale identification of cross-linked peptides Nature Communications **10**, 3404 10.1038/s41467-019-11337-z
